# Effect of *O*-linked glycosylation on the antigenicity, cellular uptake and trafficking in dendritic cells of recombinant Ber e 1

**DOI:** 10.1371/journal.pone.0249876

**Published:** 2021-04-29

**Authors:** Nuzul N. Jambari, Susan Liddell, Luisa Martinez-Pomares, Marcos J. C. Alcocer

**Affiliations:** 1 Department of Food Science, Faculty of Food Science and Technology, Universiti Putra Malaysia, Serdang, Selangor, Malaysia; 2 Laboratory of Food Safety and Food Integrity (FOSFI), Institute of Tropical Agriculture and Food Security, Universiti Putra Malaysia, Serdang, Selangor, Malaysia; 3 Division of Animal Science, School of Biosciences, University of Nottingham, Loughborough, United Kingdom; 4 School of Life Sciences, University of Nottingham, Queen’s Medical Centre, Nottingham, United Kingdom; 5 Division of Food Sciences, School of Biosciences, University of Nottingham, Loughborough, United Kingdom; Consejo Superior de Investigaciones Cientificas, SPAIN

## Abstract

Ber e 1, a major Brazil nut allergen, has been successfully produced in the yeast *Pichia pastoris* expression system as homogenous recombinant Ber e 1 (rBer e 1) with similar physicochemical properties and identical immunoreactivity to its native counterpart, nBer e 1. However, *O*-linked glycans was detected on the *P*.*pastoris*-derived rBer e 1, which is not naturally present in nBer e 1, and may contribute to the allergic sensitisation. In this study, we addressed the glycosylation differences between *P*. *pastoris*-derived recombinant Ber e 1 and its native counterparts. We also determined whether this fungal glycosylation could affect the antigenicity and immunogenicity of the rBer e 1 by using dendritic cells (DC) as an immune cell model due to their role in modulating the immune response. We identified that the glycosylation occurs at Ser96, Ser101 and Ser110 on the large chain and Ser19 on the small polypeptide chain of rBer e 1 only. The glycosylation on rBer e 1 was shown to elicit varying degree of antigenicity by binding to different combination of human leukocyte antigens (HLA) at different frequencies compared to nBer e 1 when tested using human DC-T cell assay. However, both forms of Ber e 1 are weak immunogens based from their low response indexes (RI). Glycans present on rBer e 1 were shown to increase the efficiency of the protein recognition and internalization by murine bone marrow-derived dendritic cells (bmDC) via C-type lectin receptors, particularly the mannose receptor (MR), compared to the non-glycosylated nBer e 1 and SFA8, a weak allergenic 2S albumin protein from sunflower seed. Binding of glycosylated rBer e 1 to MR alone was found to not induce the production of IL-10 that modulates bmDC to polarise Th2 cell response by suppressing IL-12 production and DC maturation. Our findings suggest that the *O*-linked glycosylation by *P*. *pastoris* has a small but measurable effect on the *in vitro* antigenicity of the rBer e 1 compared to its non-glycosylated counterpart, nBer e 1, and thus may influence its applications in diagnostics and immunotherapy.

## Introduction

*Pichia pastoris*-derived recombinant allergens have been proposed as suitable substitutes for natural allergens used in diagnostics, such as skin prick testing [1], radioallergosorbent test (RAST) [2], and protein microarray [3]. They can also be alternatives for crude extracts currently used for immunotherapy [4, 5]. The methylotrophic yeast *P*. *pastoris* expression system has been widely used in the allergy field for studies of Phl p 1, Cyn d 1 and Lol p 1 of grass group 1 allergens [6], major olive allergen, Ole e 1, and other Ole e 1-like proteins such as Fra e 1, Che a 1 or Pla l 1 [7–10]. *P*. *pastoris* is a preferred eukaryotic recombinant protein expression system over *E*. *coli* expression system because of its advantages in protein processing, folding and posttranslational modifications [11].

However, care must be taken when using recombinant allergens from fungal origin in immunotherapy since post-translational modifications may alter intrinsic features of the allergens. The observed additional glycosylation of recombinant proteins produced by *P*. *pastoris* in particular has been widely reported and reviewed elsewhere [12]. Most heterologous recombinant proteins secreted from *P*. *pastoris* are glycosylated, containing a varied number of mannose residues covalently attached to their peptide backbones [12]. In some studies, the characterised short *O*-linked saccharides of mannose containing alpha 1, 2-glycosidic linkages on *P*. *pastoris* expressed proteins did not alter their three-dimensional (3D) structures [12], whilst others reported significant differences. For instance, the hyperglycosylation on *P*. *pastoris*-derived recombinant Der p 1 hampered protein maturation, whereas the O-linked glycosylation of recombinant Dac g 5 from grass pollen allergen introduced a new epitope that differentially bound to patients IgE compared to its native counterpart [13, 14]. This hyperglycosylation thus negatively affects the sensitivity of the diagnostic tests [13, 14]. Moreover, extensive *O*-linked glycosylation of the proteins expressed by the *P*. *pastoris* system enhanced the immunogenicity of proteins by increasing the dendritic cells’ (DC) efficiency of uptake, as observed in artificially glycosylated recombinant ovalbumin [15]. The *O*-linked glycosylation generally observed in *P*. *pastoris* is not present in all recombinant allergens. As we reported previously, *P*. *pastoris*-derived recombinant 2S albumins such as rAL1 and rAL3 from soybean, and rSFA8, a recombinant 2S albumin protein from sunflower seeds, are not glycosylated [16, 17]. Therefore, an additional assessment of the glycosylation of recombinant allergens produced using fungal expression systems is required.

DC are key sentinels in the immune response and possess groups of Pattern Recognition Receptors (PRRs), such as the C-type lectin receptors (CLRs), which play important roles in sensing glycan residues on antigens. In most cases, CLR-mediated endocytosis relies on Ca^2+^ for binding to specific ligands (glycoantigens, microbes and damaged cells), and leading to the modulation of immune responses [18]. Several CLRs have been involved in the regulation of Th1/Th2 cell responses. For instance, mannose receptor (MR, CD206), DC-specific intracellular adhesion molecule-3-grabbing non integrin (DC-SIGN), Dectin-1 and Gal9 were shown to be involved in the activation of Th2 allergic response by interacting with toll-like receptor (TLR) 4 (reviewed by Salazar *et al*., 2013 [19]). The helminthic glycoprotein Schistosoma egg antigen (SEA) is recognized and internalized by human DC via a DC-SIGN and MR [20]. The glycan residue motif on Ara h 1 from peanut is suggested to act as a Th2 cell adjuvant and a ligand for DC-SIGN [21]. DC-SIGN was also shown to work in tandem with MR in DC in modulating the Th2 cell response in response to Der p 1, a major allergen from house dust mite [22]. The involvement of CLRs in the recognition of glycan residues of protein expressed in a yeast system is still poorly defined.

In a previous study we reported the overexpression of the 2S albumin model allergen recombinant Ber e 1 (rBer e 1) in the methylotropic yeast *P*. *pastoris* [16]. rBer e 1 is a recombinant form of a major allergen found in Brazil nut (*Bertholletia excelsar*). Like most 2S albumin proteins, Ber e 1 is characterised as a ~13 kDa water-soluble, heterodimeric protein that is post-translationally cleaved into two polypeptide chains with molecular weights of about 4 kDa (small subunit) and 9 kDa (large subunit) [16, 23]. It is held together by two interchain disulphide bonds between cysteine residues of large and small chains as well as two intrachain disulphide bonds on the large subunit [16, 23]. Similarities and differences between the *P*. *pastoris*-derived recombinant Ber e 1 and its native counterpart, nBer e 1 were well-reported and extensively reviewed elsewhere [16, 23, 24]. The 18 kDa precursor nBer e 1 *in planta* are processed to remove the 5 kDa N-terminal pre-pro peptide sequence, short linker (TYQT) and C-terminal flanking sequences (IAGF) into mature 12,128 Da protein that is made up of two protein subunits, 3,628 Da small subunit (Gln1 to Ser28) and 8,500 Da large subunit (Met29 to Ser102) [16, 24, 25]. While our *P*. *pastoris*-derived rBer e 1 shared the same protein sequence with nBer e 1, the rBer e 1 differs by undergoing partial post-translational processing and retaining the spacer sequence (EAEA) downstream of the Kex2 cleavage site, spacer (YQTM) and C-terminal vacuolar targeting signal sequence (IAGF) [16]. This results in a slightly larger molecular size of 13,621 Da for rBer e 1 than its native counterpart, and it is made up of 5,021 Da of small subunit (Glu1 to Arg40) and 8,600 Da of large subunit (Gly41 to Phe114) [16, 24]. On the other hand, *P*. *pastoris*-derived rSFA8, was shown to undergo further Kex2 post-translational processing into small (~5 kDa) and large (~7 kDa) subunits of polypeptides while still retaining the 3D structures and the biochemical and physical properties to its native counterpart [16, 23].

Despite sharing similar biochemical, physical and immunoreactive properties previous findings suggest that the nBer e 1 *in planta* is not glycosylated whilst rBer e 1 possesses *O*-glycans [16, 23, 26, 27]. This study therefore aimed to investigate the effect of fungal glycosylation on the *in vitro* antigenicity and immunogenicity of Ber e 1 relative to its native form. Furthermore, we also investigated whether two structurally similar plant 2S albumin proteins with different allergenic properties, Ber e 1 (glycosylated and non-glycosylated forms) and SFA8, were internalised and processed differently by bone marrow-derived (bm)DC and the effect on their maturation state.

## Materials and methods

### Media and reagents

The medium used to generate bmDC was RPMI 1640 (Gibco, UK) supplemented with 10% (v/v) heat inactivated (h.i.) foetal bovine serum (FBS) (Gibco, UK), 2mM L-glutamine (Gibco, UK), 100 μg/ml penicillin and 100 U/ml streptomycin (Gibco, UK). 2-mercaptoethanol (2-ME) (Sigma-Aldrich, UK) was used as a reducing agent in cell culture, Murine granulocyte/ macrophage colony-stimulating factor (GM-CSF) used was either *E*. *coli*-derived murine recombinant GM-CSF (mrGM-CSF) (Peprotech Rocky Hill, NJ, USA) or 15% (v/v) of conditioned medium of X63 cells producing GM-CSF (a gift from Professor Dr. William Harnett, University of Strathclyde, Glasgow, UK). Murine recombinant macrophage colony stimulating factor (mrM-CSF) was purchased from BD Biosciences. Yeast mannan from *Saccharomyces cerevisiae* (Sigma-Aldrich, Missouri, USA) was used as a C-type lectin receptor (CLR) blocker, dimethylamiloride (DMA) and rottlerin from Sigma-Aldrich were used as a macropinocytic inhibitor. DMA and rottlerin were dissolved in dimethyl sulfoxide (DMSO) at a stock concentration of 50 mM and 10 mM respectively. Alexa Flour 488 (A488) and Alexa Fluor 647 (A647) protein labelling kits were from Life Technologies. Fluorescein isothiocyanate (FITC)-Dextran (70,000 Da) (Sigma-Aldrich) was used as a macropinocytic marker. Heat-inactivated rabbit serum (Gibco, UK) was used as a blocking reagent for the flow cytometric assay.

### Antibodies

The antibodies used for phenotyping bmDC and determining their mature population are hamster phycoerythrin (PE) anti-mouse CD11c clone N418 (CD11c-PE) and its isotype control clone HTK888 from Biolegend (USA), rat Phycoerythrin-Cyanine 7 anti-mouse CD11b clone M1/70 (CD11b-PE-Cy7), hamster PE anti-mouse CD80 clone 16-10A1 (CD80-PE), rat PE anti-mouse CD86 clone GL1 (CD86-PE) and their respective isotype controls were all from BD Pharmingen (UK). ProLong® Gold Antifade Reagent with DAPI was a gift from Dr. Lee Wheldon (CBS, UoN, Nottingham, UK).

### Animal

Animals used for generating bmDC were 5-7-week-old female BALB/c strain mice, purchased from Charles River (Oxford, UK) and kept in the Sutton Bonington animal facilities. All experiments on performing tissue collection from mice were carried out under a Home Office License, in accordance with the guidelines of the UK animal (Scientific Procedures) Act, 1986. All experiments on performing tissue collection from mice were carried out post-mortem after application of a Schedule 1 humane killing procedure as specified under the UK animal (Scientific Procedures) Act, 1986. The animal study was run under the approved Home Office project PPL 40/2855/PIL 40/8088 that has been approved by the internal University of Nottingham Ethical Review Committee. Mice were euthanized by carbon dioxide asphyxiation for 6 min at a flow rate of 2 L/min and deaths were confirmed by cervical dislocation.

#### Protein expression and purification

Recombinant Brazil nut 2S albumin (rBer e 1) and sunflower seed 2S albumin (rSFA8) were produced as secreted full-length proteins by *Pichia pastoris* and purified by FPLC using a SP-Sepharose column, as described previously [23]. Total water-soluble protein and 2S albumin (nBer e 1) fractions were extracted and purified from Brazil nut kernel using a Sephacryl-S200 column, and both forms of Ber e 1 and rSFA8 were further purified by preparative reverse-phase chromatography, as previously described [16]. The reverse-phase purified rBer e 1, rSFA8, and nBer e 1 were shown to be absent of endotoxin, as determined using Pierce^®^ LAL Chromogenic Endotoxin Quantitation Kit (Thermo Scientific, UK).

#### Fluorescent-labelled 2S albumin proteins

nBer e 1, rBer e 1, and rSFA8 were chemically conjugated by an acylation process to the fluorescent Alexa Fluor 488-tetrafluorophenyl (TFP) ester and the Alexa Fluor 647-succinimidyl ester (SE) according to the manufacturer’s instructions (Life Probes, Invitrogen, UK). The concentration of allergens and the efficiency of labelling of the allergens were spectrophotometrically determined at 280 nm and 495 nm for Alexa488 (A488)-conjugants or 650 nm for Alexa647 (A647)-conjugants using Nanodrop 3300 fluorospectrometer (Nanodrop, Delaware, USA).

The degree of labelling was calculated as follows:

$$Moles\;dye\;per\;mole\;protein=\frac{A_{494}\times dilution\;factor}{71\;000\times protein\;concentration\;(M)}$$

Where 71 000 cm^-1^M^-1^ is the approximate molar extinction co-efficient of the Alexa Fluor488 dye at 494 nm.

$$Moles\;dye\;per\;mole\;protein=\frac{A_{650}\times dilution\;factor}{239\;000\times protein\;concentration\;(M)}$$

Where 239 000 cm^-1^ M^-1^ is the approximate molar extinction coefficient of the Alexa Fluor 647 dye at 650 nm.

#### Detection of glycan residues by periodic acid Schiff’s (PAS) staining

The protein samples (20 ug/well) were treated with 300 mM DTT at 95°C for 10 min and electrophoresed on a 12% Bis-Tris NuPAGE precast gel (1 mm) using the NuPAGE 12% Bis-Tris Electrophoresis System (Invitrogen, UK) according to the manufacturer’s instructions. Proteins were fixed by incubating in 50% (v/v) methanol for one hour with agitation. After two washes with deionised water for 20 min each, the gel was incubated with oxidizing agent (1% (w/v) periodic acid in 3% (v/v) acetic acid) for 2 hours with shaking, washed with deionised water two times for 20 min each, and incubated with Schiff’s reagent (Sigma-Aldrich, USA) for two hours. After removing Schiff’s reagent, the gel was incubated with reducing agent (2.5% (w/v) sodium metabisulphite in 10 mM hydrochloric acid) overnight, washed three times with deionised water to stop the reaction. An identical NuPAGE gel with samples were stained with Coomassie Brilliant Blue R-250 dye solution (0.125% (w/v) of Coomassie Brilliant Blue R250 (Sigma, USA) in 40% (v/v) methanol and 10% (v/v) acetic acid) and destained until the background is clear. Both PAS- and Coomassie-stained gels were imaged on Gel Doc XR+ System (Bio-Rad, USA).

#### Analysis of tryptic (glyco)peptides using ESI-Q-ToFII mass spectrometry (MS)

The glycosylation of rBer e 1 and nBer e 1 was confirmed by tryptic digestion of the rBer e 1 and nBer e 1 into (glyco)peptides and analysed on ESI-Q-ToFII mass spectrometry (MS). The protein samples were prepared and separated by 12% NuPAGE (Novex, USA) and stained using Coomassie colloidal blue (Sigma, UK). Protein samples were excised from gels and processed in gel pieces using the ProteomeWorks Mass PREP robotic liquid handling station (Waters, UK). The samples were then incubated three times in destain solution (50 mM ammonium bicarbonate, 50% acetonitrile) for 10 minutes each at room temperature. Afterward, the samples were dried in acetonitrile for 5 minutes and further processed by incubating in a reducing solution (10 mM dithiothreitol, 100 mM ammonium bicarbonate) for 30 minutes and, following the removal of the reducing solution, incubated in alkylation solution (55 mM iodoacetamide, 100 mM ammonium bicarbonate) for 20 minutes. The gel pieces were then washed at room temperature in 100 mM ammonium bicarbonate for 10 minutes, acetonitrile for 5 minutes and dehydrated by two washes in acetonitrile for 5 minutes and evaporated for 5 minutes. All of these processes were conducted at room temperature. The micro titre plate containing the gel plugs was cooled to 6°C for 10 minutes before adding 25 μl per well of trypsin gold (Promega, UK), and diluted to 10 ng/μl in trypsin digestion buffer (50 mM ammonium bicarbonate). The plate was incubated at 6°C for a further 20 minutes to permit trypsin entry into the gel plugs with minimal autocatalysis before being incubated at 40°C for 4 hours.

Samples were then processed; reduced, alkylated, and trypsin digested, using standard procedures. Briefly, each sample (2.5 μl) was reduced by adding 30 mM DTT in 100 mM ammonium bicarbonate. Samples were incubated for 15 minutes at 45°C. For the alkylation process, 290 mM iodoacetamide in 100 mM ammonium bicarbonate and deionised water were added to the samples, followed by incubation at room temperature in the dark for 20 minutes. The samples were then digested by adding 50 ng/μl of trypsin gold in 50 mM ammonium bicarbonate and incubated at 40°C for 4.5 hours.

As for data dependent acquisitions (DDA), LC-ESI-tandem MS on a Q-TOFII mass spectrometer fitted with a nanoflow electrospray ionization (ESI) source (Waters Ltd., UK) was used to analyse the samples. Samples were performed on a CapLC-HPLC system fitted with a PepMap C18 reverse phase, 75 μmi.d., 15-cm column (LC Packings) and the peptides were delivered on-line to the MS for 90 minutes. The mass spectrometer was operated with a capillary in positive ion mode with voltage of 3000 V, using argon as the collision gas. An automated data-dependent switching between MS and MS/MS scanning was used to acquire tandem MS data based upon mass, charge state and intensity of ion (data directed analysis (DDA^TM^)). To select different charged precursor peptide ions for fragmentation, a method was created in the MassLynx 4.0 software in which charge state recognition was used. Precursor mass was selected one at a time for acquiring tandem MS. A collision energy was chosen based on each precursor’s mass and charge and varied from 15 to 55 eV.

The non-interpreted MS data was processed into peak list (pkl) files by Protein Lynx Global Server version 2.0 (Waters, Ltd., UK). The pkl data were searched against entries in Swissprot and/or NCBInr databases using the MASCOT MS/MS ions search tool (http://www.matrixscience.com/). The predicted masses of trypsin-digested peptides of rBer e 1 and nBer e 1 were generated using the Peptide/Protein Editor software tool in MassLynx (Waters, Ltd., UK). A few modifications, such as oxidation of methionine and carbamidomethylation of cysteine, were allowed, and masses for peptides generated from multiple missed trypsin cleavages were also calculated.

The predicted mass of peptides with different numbers of mannose residue(s) attached to serine or threonine residues for each peptide were calculated. The precursor ion spectra for specific saccharide mass differences between signals in the higher-molecular mass range were then manually screened. To simplify the search for peptide masses of interest, mass spectra were deconvoluted into single charged peaks from the multiple charged signals using the MaxEnt3 algorithm (Waters, Ltd., UK). The lists of observed peptide masses were compared to the peptide masses calculated from the sequence plus or minus the known masses of variable numbers of mannose residues and correlations made where the masses matched.

#### Human DC-T cell proliferation assay

Glycosylated rBer e 1 and non-glycosylated nBer e 1 were sent to a commercial laboratory (ProImmune, Oxford, UK) and the relative antigenicity of the two proteins was analysed *in vitro* using the ProImmune REVEAL™ Immunogenicity System DC-T cell assay (Proimmune, Oxford, UK).

The assay was performed using whole proteins tested against a minimum of 40 different healthy human donor peripheral blood mononuclear cell (PBMC) samples isolated from Buffy coats including a step where CD8^+^ cells were depleted prior to use to eliminate CD8^+^ responses from the analysis. A panel of different PBMC samples from healthy human donors was selected from the ProImmune cell bank. A high starting number was chosen to ensure that the required number of donors passed all selection criteria in accordance to ProImmune standard experimental procedures. Each PBMC sample was HLA-typed by ProImmune’s PCR-sequence specific oligonucleotides (PCR-SSOP) using Luminex xMAP^®^ technology (ProImmune, Oxford, UK) and stored in liquid nitrogen (vapour-phase) prior to use. Details of HLA-DRB1, DQB1, and DPB1 alleles of donors are summarized in S2 Table. The DRB1, DQB1, and DPB1 allele frequency distributions of the donor panel in comparison to known distributions within the global population is provided in S1 Fig. Adherent PBMCs were cultured with appropriate growth factors to generate monocyte-derived DCs (moDC). At day 3, moDC cultures were loaded with either test proteins (glycosylated (rBer e 1) or non-glycosylated Ber e 1 (nBer e 1)), reference proteins (Keyhole Limpet Hemocyanin (KLH) or tuberculin purified protein derivative (PPD)) or left untreated. Test proteins were loaded at a final concentration of 50 μg/ml, as determined by their molecular weight in relation to a standard reference protein. Approximately 50–70% of donors were expected to react to KLH via a presumptive naïve immune response and 70–90% of donors to PPD through a memory immune response. Immature moDCs were cultured for another 2 days to generate mature moDCs, which were then co-cultured at a fixed ratio with autologous carboxyfluorescein succinimidyl ester (CFSE)-labelled T cells for 7 days [28]. Detection of CD4^+^ T cell proliferation was performed by co-staining with anti-human CD4 antibody. Analysis was carried out by flow cytometry (FlowJo Software, Tree Star, Inc.).

#### Data analysis

To determine antigen-induced proliferation per individual donor, data were compiled and calculated as the percentage stimulation above background. The frequency of positive donor cell responses (percentage antigenicity) was calculated by expressing responding donors out of the total donors. The values were then standardised against the strength (an average of the percentage stimulation value obtained across accepted donors for each protein) of response to give a response index (RI).

#### Determination of percentage stimulation above background

Data obtained by flow cytometric evaluation of cell samples were analysed using FlowJo Software (Tree Star, Inc.). The number of CFSE-dim (i.e. proliferating) cells was determined for each sample in 8 replicate wells. Counts for the CD4^+^ CSFE dim population in each sample were expressed as a proportion of the total CD4^+^ population. The 8 replicate values were then used to calculate the percentage stimulation above background (proportion of CD4^+^ CSFE dim cells with antigen stimulation minus the proportion of CD4^+^ CSFE dim cells without antigen stimulation). A mean and standard error of the 8 values were calculated for each sample. A one-way analysis of variance (ANOVA) was also performed on the data to determine (to a significance level of P ≤ 0.05) whether the CSFE dim population size obtained from each protein differs significantly from the untreated controls. A response that has a percentage stimulation value of 0.5% above background or greater, and is also two standard errors greater than background, is considered to be positive (this cut-off value was set by subtracting 2 x SEM from the average resting response of unstimulated cells for each donor, then taking a mean remaining percentage across all donors) extended from the mean of each direction. Data with percent stimulation value at 0.5% or greater reflects the antigenic events with greater significance identified. However, a statistically significant, but less stringent cut-off value of percent stimulation of 0.02% was also applied. Data at 0.02% of stimulation value indicated a broader-based antigenicity response profile for the selected donor population studied and are included in the final analysis.

#### Determination of percentage antigenicity

In evaluating antigenicity, the frequency of donor cell responses across the study cohort were taken into account. A positive response (percentage stimulation above background ≥ 0.5%) in 2 or more independent donor samples indicates a potential T cell epitope. In this study with a sample size of 40 donors, two responding donors set a threshold of 5%. Following the calculation of percentage of stimulation above the background values, the frequency of positive donor cell responses (percentage of antigenicity) was expressed as the proportion of responding donors out of total accepted donor screened (Proimmune, Oxford, UK).

#### Response index (RI)

The strength of positive donor cell responses was determined by calculating the average of the percentage stimulation value obtained across accepted donors for each protein. Determining a response index (RI) through the multiplication of strength and frequency values represents more of the level of antigenicity, as opposed to methods of analysis that rely on the frequency of response alone, as some may be pan-allele binding (Proimmune, Oxford, UK).

$$RI=\frac{\left(mean\;\%\;donors\;responding)\times (average\;strength\;of\;response\right)}{100}$$

The *in vitro* antigenicity of each protein was assessed by measuring both the strength and frequency of donor cell responses. Percentage stimulation above background was determined for each donor and the frequency of positive donor responses (percentage antigenicity) was calculated by expressing responding donors out of total donors. Values were then standardised against the strength of response (an average of the percentage stimulation value obtained across accepted donors for each protein) to give a response index (RI). The percentage of antigenicity was measured for each donor and the responding donors that gave a positive response above background (with strength response cut-offs of 0.02% and 0.5%) were determined. Any positive response observed in a donor sample indicated that a proliferative immune response has been mounted on at least one of the six HLA class alleles presented by that specific donor.

#### Generation of murine bone marrow-derived dendritic cells (bmDC)

Murine bmDC were then generated as described by Kean *et al*. (2006) with slight modifications. Animals used for generating bmDC were 5-7-week-old female BALB/c strain mice, purchased from Charles River (Oxford, UK) and housed according to the laboratory animal housing conditions of the University of Nottingham Sutton Bonington facility, which followed the DEFRA requirements. All experiments on performing tissue collection from mice were carried out post-mortem after application of a Schedule 1 humane killing procedure as specified under the UK animal (Scientific Procedures) Act, 1986. The animal study was run under the approved Home Office project PPL 40/2855/PIL 40/8088 that had been approved by the internal University of Nottingham Ethical Review Committee. Mice were euthanized by CO_2_ asphyxiation for 6 min at a flow rate of 2 L/min and death was confirmed by cervical dislocation. Femurs and tibias of mice were dissected, pooled, sterilized with 70% (v/v) ethanol for 1 min, and then washed with RPMI 1640 medium supplemented with 100 units/ml of Streptomycin and 100 μg/ml of Penicillin. The epiphyses of the bones were cut and the bone marrows were flushed out using sterile 25 G needle attached to 20 ml syringe containing complete R10 medium (RPMI 1640 medium (Gibco, UK) and supplemented with 10% (v/v) heat-inactivated FBS (Gibco, UK), 2mM L-glutamine, 100 U/ml of penicillin, 100 μg/ml of streptomycin). The bone marrow was centrifuged at 235 × g, 4°C for 5 min. Supernatant was removed and cells were resuspended in a complete R10 medium by pipetting harshly to disintegrate the bone marrow cell aggregates. The bone marrow cells were then cultured in a 100 mm × 20 mm tissue culture-treated petri dish (Corning, USA) in a complete R10 medium (RPMI1640 10% (v/v) FBS supplemented with 5 μM beta-mercaptoethanol (2-ME) and 20 ng/ml of recombinant murine GM-CSF (rmGM-CSF) (Peprotech, USA)), and then incubated at 37°C, 5% CO_2_ for 8 days. The medium was replaced with complete R10 medium on days 3 and 6 by gentle aspiration. On day 8, loosely adherent cells were harvested and pooled as immature DC. The total percentage of population of CD11c^+^ DC was determined by flow cytometry (see below).

#### Phenotyping the bmDC surface markers

The cell populations of bmDC were analysed by measuring CD11c^+^ and CD11b^+^ populations using flow cytometry. The phenotypes of mature bmDC were analysed after stimulating cells with either 50 μg/ml of nBer e 1, rBer e 1 or rSFA8 or 10 ng/ml of LPS for 24 h. The level of cell surface co-stimulatory molecules, CD80, and CD86, were measured by flow cytometry using Beckman Coulter FC500 flow cytometer (Beckman Coulter, UK). Non-stimulating bmDC were used as a control. Aliquots of 1 × 10^6^ cells/ml of bmDC (both untreated and treated with nBer e 1, rBer e 1, rSFA8 and LPS for 24 hours) were centrifuged at 350 x g, at 4°C for 5 minutes and the supernatants were discarded. Cells were washed three times with cold fluorescence-activated cell sorting (FACS) wash buffer (2.5% (w/v) bovine serum albumin (BSA), 50 μM EDTA (pH 8.0), 100 μM sodium azide (NaN_3_) in 1 x PBS) and then re-suspended in FACS blocking buffer medium (2.5% (v/v) heat-inactivated rabbit serum in FACS wash buffer) with 2.4G2 (anti-FcγRII/III) antibody at 1:1000 dilution and incubated on ice for 30 minutes. Then, 50 μl of 100 000 pre-chilled cells were seeded on a pre-chilled V-bottom non-tissue culture treated 96 well plate (Falcon Scientifics, USA). Fifty microlitre of 2.5 μg/ml of flourophore-conjugated antibodies (CD11b-PE-Cy7, CD11c-PE, CD80-PE, CD86-PE, or their respective isotype controls in FACS) wash buffer were added to the cells and incubated on ice for an hour in the dark. The cells were then centrifuged at 350 × g for 5 minutes at 4°C and the supernatants were removed. Cells were washed 3 times with FACS wash buffer and fixed with 2% (v/v) formaldehyde in 300 μl of FACS wash buffer for flow cytometric analysis using Beckman Coulter FC500 flow cytometer (Beckman Coulter, UK). Data were then analysed using Weasel V3.0.2 Australia).

#### *In vitro* murine bmDC uptake assay

For the *in vitro* uptake of fluorescent-labelled proteins by bmDC, 5.0 x 10^5^ cells/ml of bmDC in a serum-free X-vivo 10 media without phenol red (Lonza, UK) were conditioned for 30 minutes at 37°C, 5% CO_2_. For inhibition assay, bmDC were pre-treated with different concentrations of dimethyl amiloride (DMA), rottlerin, and mannan for 30 minutes at 37°C, 5% CO_2_. Yeast mannan from *Saccharomyces cerevisiae* (Sigma-Aldrich, Missouri, USA) was used as a C-type lectin receptors (CLR) blocker, while dimethylamiloride (DMA) and rottlerin from Sigma-Aldrich, UK, were used as a macropinocytic inhibitor. DMA was dissolved in DMSO at a stock concentration of 50 mM. Culture medium was removed and cells were pulsed with fluorescent-labelled proteins or dextran-FITC in X-vivo 10 and further incubated at 37°C and 5% CO_2_ for 45 minutes. Cells were collected by centrifugation at 350 x g, 4°C for 5 minutes and incubated in ice-cold FACS block solution (0.5% (v/v) heat inactivated rabbit serum, 0.5% (w/v) BSA, 100 μM NaN_3_, 100 μM EDTA in PBS (pH 7.2)) with anti-FcγR II/III—monoclonal antibody 2.4G2 (rat IgG2b kappa), at a 1:1000 dilution, for 30 min on ice. PE-conjugated hamster anti-mouse CD11c (2.5 μg/ml) was added to the cells and cells were further incubated for an hour. Cells were washed three times with FACS solution (0.5% (w/v) BSA, 100 μM NaN_3_, 100 μM EDTA in PBS (pH 7.2)), fixed with 2% (v/v) paraformaldehyde (PFA) in FACS solution and analysed on Beckman Coulter FC500 (Beckman Coulter, UK). Twenty thousand events were collected for each sample with appropriate filter combinations. Data analysis was performed using Weasel 3.2.0 software (WEHI, Australia). CD11c^+^ bmDC population on a dot plot graph of forward scatter (FS linear) versus side scatter (SS linear) was gated and assigned as region for analysis.

#### CHO and CHO-MR endocytosis assay

CHO and CHO-MR endocytosis assay was performed according to method by Su *et al*. (2005) [29]. Two hundred thousand cells per millilitre of Chinese hamster ovarian cell line (CHO) and stable CHO cell line transfected with murine MR (CHO-MR) were seeded in a complete DMEM F-Ham 12 medium supplemented with 10% (v/v) h.i. FBS in a 24-well plate overnight at 37°C, 5% CO_2_. Cells were washed with Dulbecco’s phosphate buffered saline (DPBS) (Gibco, UK) twice and conditioned in OPTI-MEM medium (Gibco, UK) for 30 min, 37°C, 5% CO_2_. Culture medium culture was aspirated and cells were pulsed with 10 μg/ml of labelled proteins in OPTI-MEM for an hour at 37°C, 5% CO_2_. Cells were harvested by treating with trypsin-EDTA and fixed in 2% (v/v) PFA in DPBS. Cells were analysed on a Beckman Coulter FC500 and data analysis performed using Weasel 3.2.0 software (WEHI, Australia).

#### Colocalization analysis by confocal imaging

The bmDC (2.0 x 10^6^ cells/ml) in serum free X-vivo 10 without phenol red (Lonza, UK) medium were pre-incubated on a UV-sterilized V-bottom, non-tissue cultured 96-well plate (Falcon Scientifics, USA) at 4°C for an hour. Cells were pulsed with 50 μg/ml of each combination of rBer e 1-A488 (green) and nBer e 1-A647 (red), rBer e 1-A488 and rSFA8-A647, and nBer e 1-A488 and rSFA8-A647 for 30 min. After pulsing with the labelled proteins, bmDC were washed three times with ice-cold X-vivo 10 medium and fixed immediately (t = 0 minutes) or chased for another 15, 45, or 120 minutes by incubating at 37°C, 5% CO_2_ and fixed with 4% (v/v) PFA and 0.1% (v/v) glutaraldehyde in PBS for 10 min. Fixed cells were then washed three times with ice-cold PBS, centrifuged at 350 × *g*, 4°C for 5 minutes, and 95% of the supernatant were removed. Concentrated, fixed cells were mounted on a 0.16 mm thickness and 12 mm in diameter of coverslip with Prolong Gold Anti-fade mountant with DAPI (Life Technologies). Imaging was performed on a Leica TCS SP5 X Supercontinuum Confocal Microscope (Leica, Germany) at Central Laser Facilities (CLF), Science and Technology Facilities Centre (STFC), UK. Images were acquired as a single plane using a 100 ×/1.40 plan-apochromat oil objective (NA 1.4) and the appropriate filter combinations: Pinhole was set at 1.0 airy units (A.U.), zoom factor at 1.50, speed at 400 Hertz (Hz), phase X at -28.99, pixel size at 75.76 nm x 75.76 nm, and optical section at 0.896 μm. The background image was subtracted from the filtered image to obtain a background-subtracted quotient image using a ’rolling ball’ algorithm of Fiji ImageJ [30, 31]. Colocalization was quantified using BioimageXD. A binary image stack, or ’mask’, was created by thresholding from the background images using Costes randomization test [32]. This mask represents the area in which both fluorescent-labelled proteins colocalized. Mander’s colocalization coefficients (MCC) were then calculated to estimate the degree of colocalization [33]. MCC >0.8 indicates very strong colocalization, 0.6–0.8 as strong, 0.4–0.6 as medium, and <0.4 as weak colocalization [34].

$$MCC=\frac{\sum_{i}Gi,coloc}{\sum_{i}Gi},\;\mathrm{where}\;G_{i,coloc}=G_{i}\;if\;R_{i}>0\;and\;G_{i,coloc}=0\;if\;R_{i}=0$$

#### Measurement of Th1/Th2 cytokines

Luminex assay was performed using a commercial kit, mouse Th1/Th2 6-plex panel (Invitrogen, Life Technologies, USA) to measure the levels of IL-2, IL-4, IL-5 and IL-10 (Th2 determining cytokines), and IL-12 and IFN-γ (Th1 determining cytokines). The supernatant from 1 x 10^5^ cells/ml of bmDC stimulated with 50 μg/ml of nBer e 1, rBer e 1, rSFA8 or 5 μg/ml of LPS as a control for 24 h were analysed on Luminex Detection System using Bioplex 200 System (Bio-Rad Laboratories Inc., UK). Details of the concentration of standards for each cytokine and their respected bead regions were entered into the system for analysis. Data were analysed using BioPlex Manager (Bio-Rad Laboratories Inc., UK) to retrieve fluorescence intensity (FI) readings of R-Phycoerythrin (R-PE) labelled standards and samples. The 5-Parametric logistic equation was used to calculate the concentration of cytokines in each sample.

#### Statistical analysis

Statistical significances of data were calculated using one-way ANOVA or two-way ANOVA in Graphpad Prism 5.0. For one-way ANOVA analysis, Tukey’s test was performed as *posthoc* analysis.

## Results

### *P*. *pastoris*-expressed recombinant Ber e 1 is heterogeneously glycosylated

**Fig 1:**
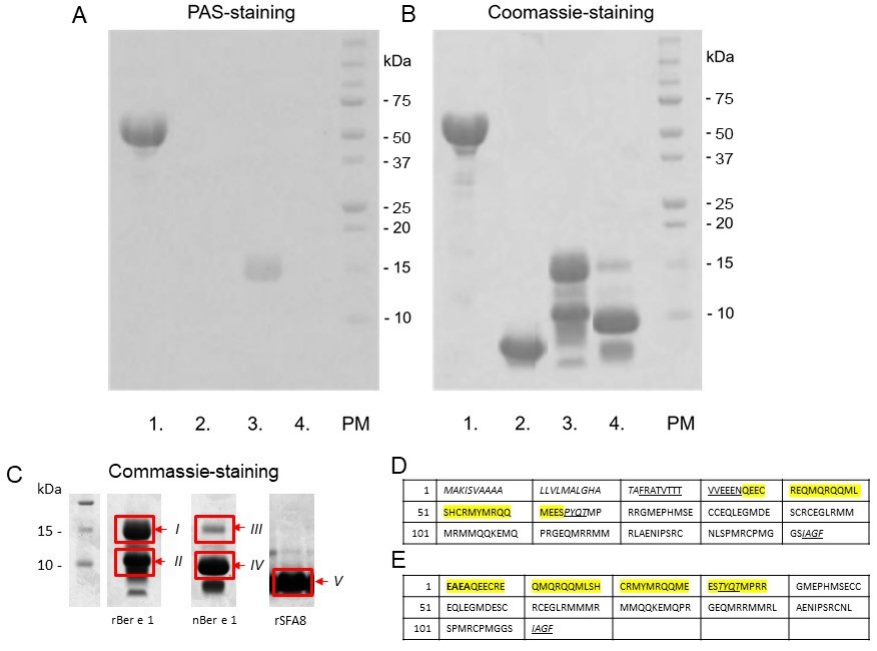
Detection of glycosylation on 2S albumin proteins by periodic acid Schiff’s (PAS) staining. (A.) A positive glycoprotein control, CPDY (1.), rSFA8 (2.), rBer e 1 (3.), and nBer e 1 (4.) were separated on a 12% BisTris NuPAGE gel under denaturing conditions (300 mM DTT) and PAS stained to detect glycoproteins. Only the ~15 kDa band of rBer e 1 and the positive control, CPDY, reacted with the PAS stain, indicating the presence of glycan residues. nBer e 1 and rSFA8 did not react with PAS stain, thus indicate the absence of glycosylation according to this assay. (B.) An identical NuPAGE gel stained with Coomassie brilliant blue shows all of the proteins present. Raw images of Commassie- and PAS-stained gels are presented in (S3 Fig). (C.) rBer 1, nBer e 1 and rSFA8 protein bands were excised for peptide identification. Samples of rBer e 1(*I* and *II*), and nBer e 1 (*III* and *IV*) and rSFA8 (*V*) were trypsinised and analysed by LC-MSMS analysis. (D.) Amino acid sequence of precursor native Ber e 1 *in planta*. Italic fonts represent the N-terminal signal sequence, the underlined fonts represent the pre-prosignal sequences, and the italic and underlined fonts represent the short linker and C-terminal linker. These sequences were cleaved and removed to produce mature Ber e 1 *in planta* that consists of small subunit (yellow highlight) and large subunit. (E.) Amino acid sequence of mature recombinant Ber e 1 retained the spacer sequence (bold fonts) downstream of the Kex2 cleavage site, spacer, and C-terminal vacuolar targeting signal sequence (italic and underlined fonts) and it is made up of small subunit (yellow highlight) and large subunit [16, 24].

In order to confirm whether glycan residues are present on the model proteins rBer e 1, nBer e 1, and rSFA8, NuPAGE-Periodic Acid Schiff’s (PAS) staining was carried out (Fig 1). Based on SDS-PAGE-PAS staining of reduced proteins, only the large band of rBer e 1 at 15 kDa was glycosylated whereas its wild-type counterpart, nBer e 1, and rSFA8, were not. When compared with the glycoprotein carboxypeptidase Y (CPDY) (Fig 1, band 1), the lower intensity of PAS staining on 15 kDa band of rBer e 1 corresponded to the presence of short mannose residues found on rBer e 1 produced by *P*. *pastoris*.

The glycosylation binding site and the possible profile of mannose moieties attached to rBer e 1 were determined by analysing peptides from tryptic digested rBer e 1 and nBer e 1 using ESI-Q-ToFII MS. In-gel trypsin digestion was performed on proteins that were separated by 12% BisTris NuPAGE under reducing conditions (Coomassie stained gel B on Fig 1) and the resulting peptide mixtures were analysed by nanoLC-ESI-Q-ToFII MS. The predicted mass of peptides with different numbers of mannose residue(s) attached to serine or threonine residues were calculated. The proposed matches of peptides and glycopeptides from each sample were summarized in Table 1.

**Table 1:** Comparison and correlation of observed masses with calculated masses of tryptic peptides of rBer e 1 and nBer e 1.

Peptide sequence*	Amino acid residues	Modifications			Calculated mass	Detected mass
		Oxidation	Carbamidomethylation (CAM)	Mannose residue		
**Band I (Sample rBer e 1 (15 kDa))**						
CNL**S**PMR	98–104	-	-	6 mannose on Ser	1792.38	1792.27
LAENIP**S**R	90–97	-	-	-	899.5	899.49
LAENIP**S**R	90–97	-	-	5 mannose on Ser	1709.5	1709.02
C^CAM^PMGG**S**IAGFE	105–115	-	1 CAM on Cys	1 mannose on Ser	1287.45	1287.32
CPM^OX^GG**S**IAGFE	105–115	1 oxidation on Met	-	6 mannose on Ser	2056.45	2056.19
M^OX^MRLAENIP**S**R	87–97	1 oxidation on Met	-	2 mannose on Ser	1657.68	1657.52
LAENIP**S**RCNL**S**PMR	90–104	-	-	4 mannose on Ser	2348.86	2348.62
LAENIP**S**RC^CAM^NL**S**PM^OX^R	90–104	1 oxidation on Met	1 CAM on Cys	6 mannose on Ser	2745.86	2745.61
CNLSPM^OX^RCPMGGSIAGFE	98–115	1 oxidation on Met	-	-	1885.81	1885.03
C^CAM^NLSPM^OX^RCPM^OX^GGSIAGFE	98–115	2 oxidation on Met	1 CAM on Cys	1 mannose on Ser	2120.81	2120.31
C^CAM^NLSPM^OX^RC^CAM^PM^OX^GGSIAGFE	98–115	1 oxidation on Met	2 CAM on Cys	1 mannose on Ser	2161.81	2161.42
CNLSPM^OX^RCPM^OX^GGSIAGFE	98–115	2 oxidation on Met	-	2 mannose on Ser	2225.81	2225.44
GM^OX^EPHM^OX^SEC^CAM^C^CAM^EQLEGM^OX^DESCR	41–61	3 oxidation on Met	2 CAM on Cys	-	2644.87	2644.74
QQML**S**HCR	15–22	-	-	5 mannose on Ser	1812.46	1812.32
QQM^OX^L**S**HCR	15–22	1 oxidation on Met	-	5 mannose on Ser	1828.46	1828.31
QQML**S**HCRMDMR	15–26	-	-	1 mannose on Ser	1697.67	1697.25
QQM^OX^L**S**HC^CAM^RMDMR	15–26	1 oxidation on Met	1 CAM on Cys	1 mannose on Ser	1770.67	1769.15
QQM^OX^L**S**HC^CAM^RMDMR	15–26	1 oxidation on Met	1 CAM on Cys	2 mannose on Ser	1932.67	1932.26
QQM^OX^L**S**HC^CAM^RM^OX^DMR	15–26	2 oxidation on Met	1 CAM on Cys	4 mannose on Ser	2272.67	2272.47
QQM^OX^L**S**HC^CAM^RM^OX^DM^OX^R	15–26	3 oxidation on Met	1 CAM on Cys	5 mannose on Ser	2393.67	2392.71
EQM^OX^QRQQML**S**HC^CAM^R	10–22	1 oxidation on Met	1 CAM on Cys;	1 mannose on Ser	1909.76	1909.23
**Band II (Sample rBer e 1 (12 kDa))**						
QQMLSHC^CAM^RMDMR	16–26	-	1 CAM on Cys	-	1592.67	1592.63
RMMR	86–90	-	-	-	593.3	593.16
M^OX^MQQK	71–75	1 oxidation on Met	-	-	681.31	681.23
LAENIPSR	90–97	-	-	-	899.5	899.48
**Band III (Sample nBer e 1 (15 kDa))**						
EQMQRQQMLSHC^CAM^R	6–18		1 CAM on Cys	-	1592.67	1592.63
RMMR	77–80	-	-	-	593.3	593.16
M^OX^MQQK	62–66	1 oxidation on Met			681.31	681.24
LAENIPSR	81–88	-	-	-	899.5	899.49
**Band IV (Sample nBer e 1 (12 kDa))**						
MMR	78–80	-	-	-	437.2	437.2
LAENIPSR	81–88	-	-	-	899.5	899.49

*^CAM^ carbamidomethylation of cysteine; ^OX^ oxidation of methionine.

The MS data in Table 1 show several rBer e 1 peptide peaks with masses correlating with predicted masses of putative glycosylated peptides. The data suggest that glycosylation was present on the large subunit of the rBer e 1 with the main amino acid residues involved being: Ser101 (up to 6 mannose residues), Ser096 (up to 5 mannose residues), and Ser110 (up to 6 mannose residues). These results are in agreement with the SDS-PAGE and PAS staining results and are also in agreement with previous NMR analysis of rBer e 1 [26]. Further detected masses of (glyco)peptides suggested that the Ser19 on the small subunit of rBer e 1 was also glycosylated with up to 5 mannose residues. Overall, the results of PAS-staining SDS-PAGE and MS data corroborate previous findings describing nBer e 1 as non-glycosylated proteins *in planta* [35] like the majority of 2S albumin seed proteins, and is in agreement with our previous findings as well [16, 26]. The nBer e 1 was thus used as the non-glycosylated form of the Ber e 1 protein model.

We further confirmed the identities of the proteins from rBer e 1 and nBer e 1 samples by MS/MS peptide analysis. The total purified protein fractions of rBer e 1, nBer e 1 and rSFA8 were separated on SDS-PAGE gel and visualised by staining with Coommassie Blue, as shown in Fig 1B. Tandem MS/MS mass spectral analyses of peptide fragments excised from the gel band I and II (rBer e 1), band III and IV (nBer e 1), and band V (rSFA8) from Fig 1C were summarised in Table 2. The MS/MS spectra of the tryptic peptides with ion scores higher than 46 are indicated with 95% confidence). The MS/MS spectra lower than 46 were inspected manually. Details of the MS/MS fragmentation of the tryptic peptides for each protein band were tabulated in S1 File (excel file on MSMS data).

**Table 2:** Peptide sequence identified by ESI-Q-ToFII MS and MASCOT database search and de novo sequencing by Mass Lynx software.

Sample	Excised band (kDa)	Protein identification	Accession #	Total score	Theoretical molecular weight/ pI	Protein chain (S/L)*	Peptide sequence	Sequence coverage (%)
Band I (rBer e 1)	15	Chain A, Solution structure of the Brazil nut 2S Albumin Ber e 1	gi|409106965	183	13621/ 6.19	S	^**1**^**EAEAQEECREQMQR**^**14**^	65
						S	^**15**^**QQMLSHCR**^**22**^	
						S	^**23**^**MYMRQQMEESTYQTMPR**^**39**^	
						S	^**41**^**GMEPHMSECCEQLEGMDESCR**^**61**^	
						L	^**90**^**LAENIPSR**^**97**^	
						L	^**98**^**CNLSPMR**^**104**^	
Band II (rBer e 1)	9	Chain A, Solution structure of the Brazil nut 2S Albumin Ber e 1	gi|409106965	154	13621/ 6.19	L	^**41**^**GMEPHMSECCEQLEGMDESCR**^**61**^	31
						L	^**90**^**LAENIPSR**^**97**^	
						L	^**98**^**CNLSPMR**^**104**^	
Band III (nBer e 1) ǂ	15	2S albumin [*Bertholletia excelsa*]	gi|8439533	N/A	N/A	S	^**11**^**EQMQRQQMLSHCR**^**22**^	20
						L	^**86**^**RMMR**^**89**^	
						L	^**71**^**MMQQK**^**75**^	
						L	^**90**^**LAENIPSR**^**97**^	
Band IV (nBer e 1)	9	2S albumin [*Bertholletia excelsa*]	gi|8439533	172	16898/ 5.96	L	^**73**^**GMEPHMSECCEQLEGMDESCR**^**93**^	26
						L	^**102**^**MMQQKEMQPR**^**112**^	
						L	^**90**^**LAENIPSR**^**97**^	
Band V (rSFA8)	5	Full Albumin 8 [*Helianthus annuus*]	gi|112745	86	16078/ 5.35	L	^**87**^**QLCCMQLK**^**94**^	23
						L	^**117**^**MRDQVMSMAHNLPIECNLMSQPCQM**^**141**^	

*S Small subunit, L Large subunit

ǂ manual comparison of spectra with calculated spectra from NCBI database (Accession number gi|8439533).

The 15-kDa gel band (band I) was shown to have peptide fragments of ^1^EAEAQEECREQMQR^14^, ^15^QQMLSHCR^22^, and ^23^MYMRQQMEESTYQTMPR^39^ from the small subunit and ^41^GMEPHMSECCEQLEGMDESCR^61^, ^90^LAENIPSR^97^ and ^98^CNLSPMR^104^ from the large subunit of Ber e 1 while the 9-kDa gel band (band II) was shown to have peptide fragments of the large Ber e 1 subunit only (^41^GMEPHMSECCEQLEGMDESCR^61^, ^90^LAENIPSR^97^, and ^98^CNLSPMR^104^). We were not able to confirm the peptide identities of Band III of nBer e 1 sample by MS/MS but based on peptide mass matching, the band indicated the presence of peptide sequences of ^11^EQMQRQQMLSHCR^22^ from the small subunit, and ^86^RMMR^89^, ^71^MMQQK^75^, and ^90^LAENIPSR^97^ from the large subunit. Only the peptide fragment with amino acid sequences of ^73^GMEPHMSECCEQLEGMDESCR^93^, ^102^MMQQKEMQPR^112^, ^90^LAENIPSR^97^ from the large subunit of the Ber e 1 was identified in the 9 kDa band IV of nBer e 1. We were also able to identify peptide fragments of ^87^QLCCMQLK^94^ and ^117^MRDQVMSMAHNLPIECNLMSQPCQM^141^ in the 5-kDa band V of rSFA8 that correspond to the 2S albumin 8 from sunflower seed. This MS/MS data further confirmed that the 15 kDa bands (band I and band III) from both rBer e 1 and nBer e 1 consist of small and large subunits of the Ber e 1 protein while 9 kDa gel bands (band II and IV) only consist of large subunit of the Ber e 1 protein only.

### *In vitro* antigenicity assessment of rBer e 1

**Fig 2:**
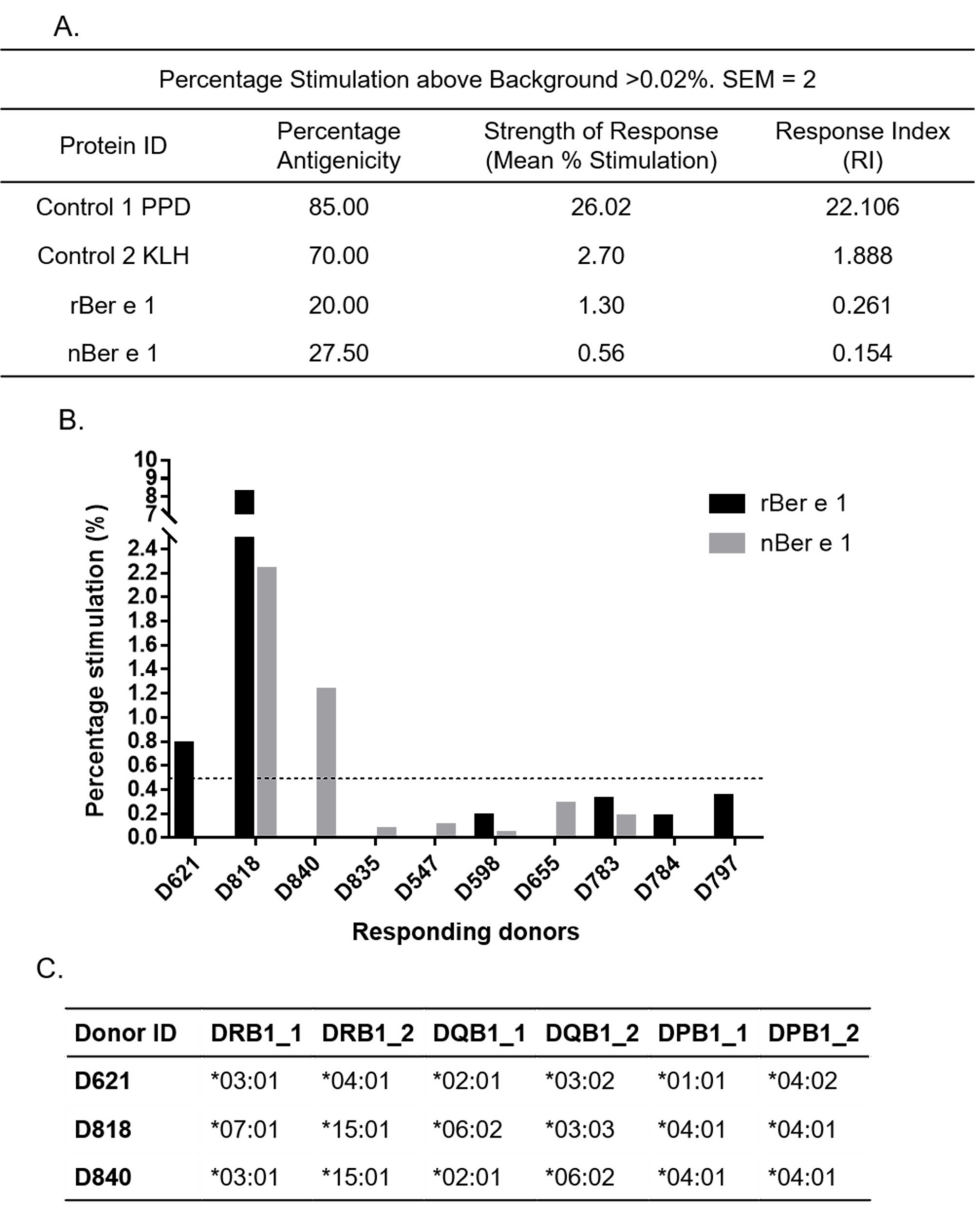
*In vitro* antigenicity of rBer e 1 and nBer e 1 by DC-T cell response assay. (A.) Response Index (RI) values for rBer e 1, nBer e 1 and reference protein from percentage antigenicity and stimulation data (percent stimulation above background ≥ 0.02%, SEM = 2) (B.) Graph of responding donors with percentage stimulation above 0.02% (SEM = 2) after stimulating with nBer e 1 and rBer e 1. Dotted line indicates higher stimulation cut-off of 0.5% (C.) Table of DRB1, DQB1, and DPB1 allele profiles of donors responding at the higher stimulation cut off of ≥0.5% (SEM = 2) when stimulated with rBer e 1 or nBer e 1. Percentage stimulation above this cut-off indicates a greater significance of antigenic response.

Whether the fungal *O*-linked glycosylation affects the antigenicity of the recombinant Ber e 1 was assessed using a commercial ProImmune REVEAL™ Immunogenicity System DC-T cell assay. The donor cohort used in this study was selected so that, as far as was possible, the most highly expressed HLA class II alleles in the global population were well represented (refer to S2 Table and S1 Fig). The HLA class II molecules determine the sequence of the peptides that can be bound and presented to the T cells [36]. Any protein that elicited responses from two or more independent donors i.e. at least 5% antigenicity (where percentage stimulation above background is equal or more than 0.5%) is considered indicative of a potential *in vivo* T cell response. Broader-based antigenicity response profiles for selected donors were also compared between nBer e 1 and rBer e 1 (where percentage stimulation above background was ≥ 0.02%, SEM = 2. Whilst less stringent this is still statistically significant). When the most stringent cut-off for percentage stimulation was applied (percentage stimulation above background ≥0.5%, SEM = 2) the percentage antigenicity for rBer e 1 and nBer e 1 were 5% and 10% respectively and the mean percentage stimulation is 4.5% and 1.19%, giving response indexes (RIs) of 0.225 and 0.119. Using the lower stimulation threshold, the percentage antigenicity rose to 20% and 27.5% respectively, however the mean percentage stimulations fell to 1.3% and 0.56%, giving RIs of 0.261 and 0.154 (Fig 2A and 2B). Although both forms of Ber e 1 had very low RIs compared to positive controls, rBer e 1 had a higher RI. These results indicate that both rBer e 1 and nBer e 1 are not strongly antigenic but potentially capable of eliciting T cell responses.

When the profile of donors that elicit antigenic response to rBer e 1 and nBer e 1 with percentage stimulation ≥ 0.02%, SEM = 2 was determined, differences in the frequency of donors with different HLA phenotypic background were observed (Fig 2B). In total, 10 donors were shown to give response when stimulated with either rBer e 1 or nBer e 1 but only 3 donors, D818, D783 and D598, were responsive to both glycosylated rBer e 1 and non-glycosylated nBer e 1. This data suggests that there may be varying degrees of antigenicity elicited by rBer e 1 and nBer e 1 and both proteins bind to different combination of HLAs at different frequencies.

### Glycosylation of the recombinant Ber e 1 increased the rate of uptake by bmDC

**Fig 3:**
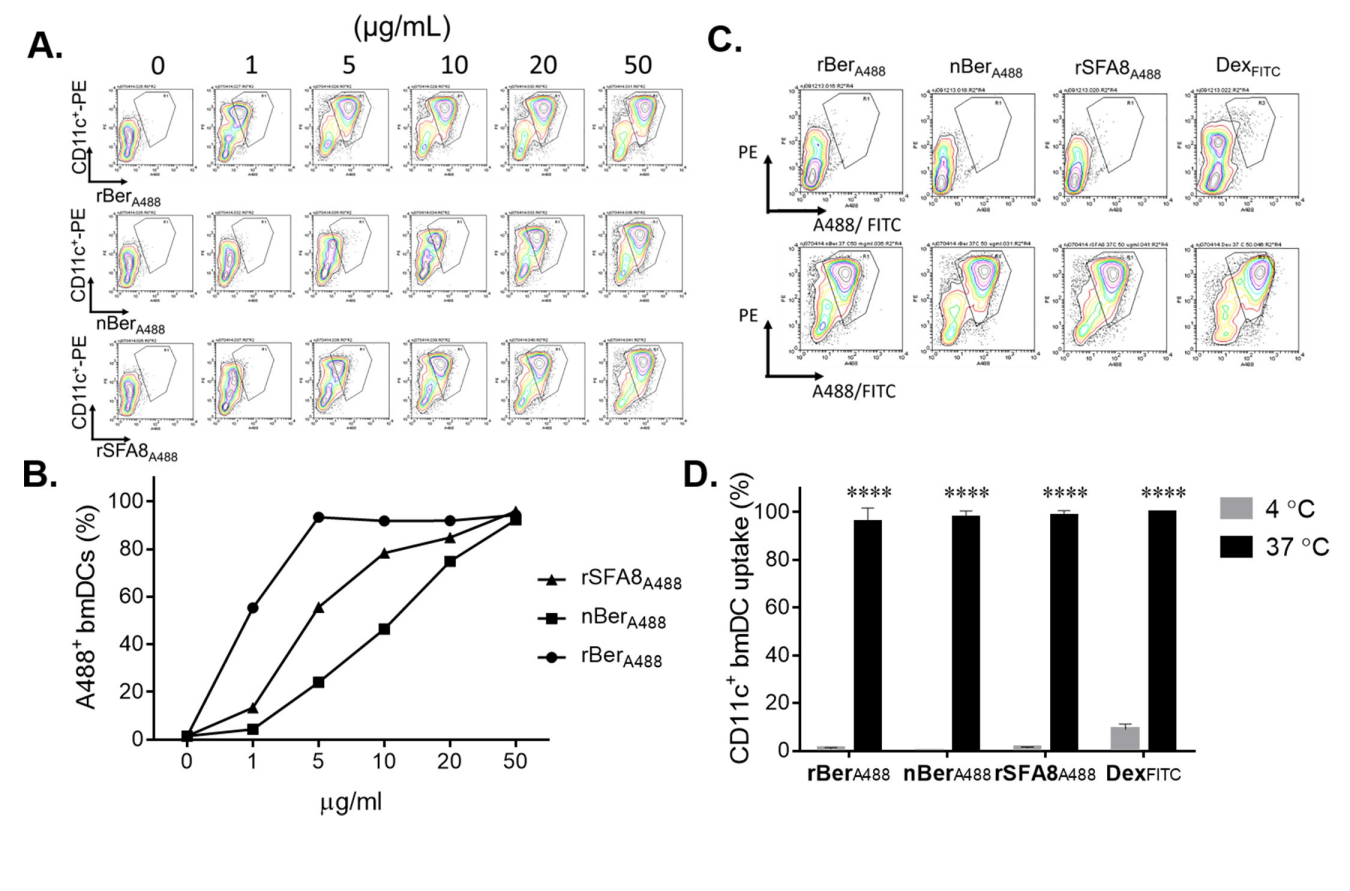
Uptake of glycosylated Ber e 1 and non-glycosylated Ber e 1 and SFA8 by bmDC. (A.) The contour plots represent cell populations where cells in the gating area are CD11c^+^ bmDC with A488-labelled proteins. DC uptake was performed with increasing concentration (1–50 μg/ml) of A488-labelled 2S albumin proteins for 45 min (B.). Data were normalised as a percentage of A488^+^ and PE^+^ gated cells relative to the untreated control. (C.) The contour plot showed the distribution of CD11c^*+*^/A488^*+*^ or FITC^*+*^ cell population in the gated region. Proteins were internalised as shown when bmDC were pulsed for 45 min with labelled proteins at 4°C (cell-surface bound) and 37°C (internalised). Data were normalised to a percentage of the A488/FITC^*+*^ bmDC population incubated with Dextran-FITC at 37°C and are presented as mean ±S.D. (n = 2) ****p<0.0001 (D.).

The effect of glycosylation of the rBer e 1 on its uptake rate by murine bmDC was investigated (Fig 3). 96.61% of the generated GM-CSF-induced bmDC that were used in this study are CD11c^+^ population (shown in the S2 Fig). The uptake of fluorescent-labelled proteins, rBer e 1-A488, nBer e 1-A488, and rSFA8-A488, by bmDC was measured by flow cytometry. The degree of labelling of proteins labelled with Alexa-488 dye was comparable, where rBer e 1-A488 is 0.20 moles dye per mole of rBer e 1, nBer e 1-A488 is 0.37 moles dye per mole of nBer e 1, and rSFA8-A488 is 0.22 moles dye per mole of rSFA8. Glycosylated rBer e 1-A488 at 5 μg/ml was taken up at rates of 2-fold and 5-fold higher than nBer e 1-A488 and rSFA8-A488 (50% CD11c). At 50 μg/ml, all rBer e 1-A488, nBer e 1-A488 and rSFA8-A488 showed a consistent rate of uptake by bmDC (Fig 3A and 3B). For the subsequent uptake analysis, 50 μg/ml was used for each protein. Whether the uptake of rBer e 1, nBer e 1 and rSFA8 occurred via energy dependent process was investigated by comparing uptake at 4 and 37°C [37]. bmDC were cultured with rBer e 1-A488, nBer e 1-A488, or rSFA8-A488 for 45 minutes, and the number of CD11c^+^ bmDC with internalized fluorescent-labelled proteins were assessed by flow cytometry (Fig 3C and 3D). Uptake of the fluorescent-labelled proteins, including dextran-FITC as control, by bmDC were reduced by more than 90% at 4°C in comparison to bmDC at 37°C, indicating that the protein was internalised into the bmDCs instead of binding to the cell surface.

### Comparison of the routes of endocytosis by bmDC for glycosylated rBer e 1 and non-glycosylated nBer e 1 and rSFA8

**Fig 4:**
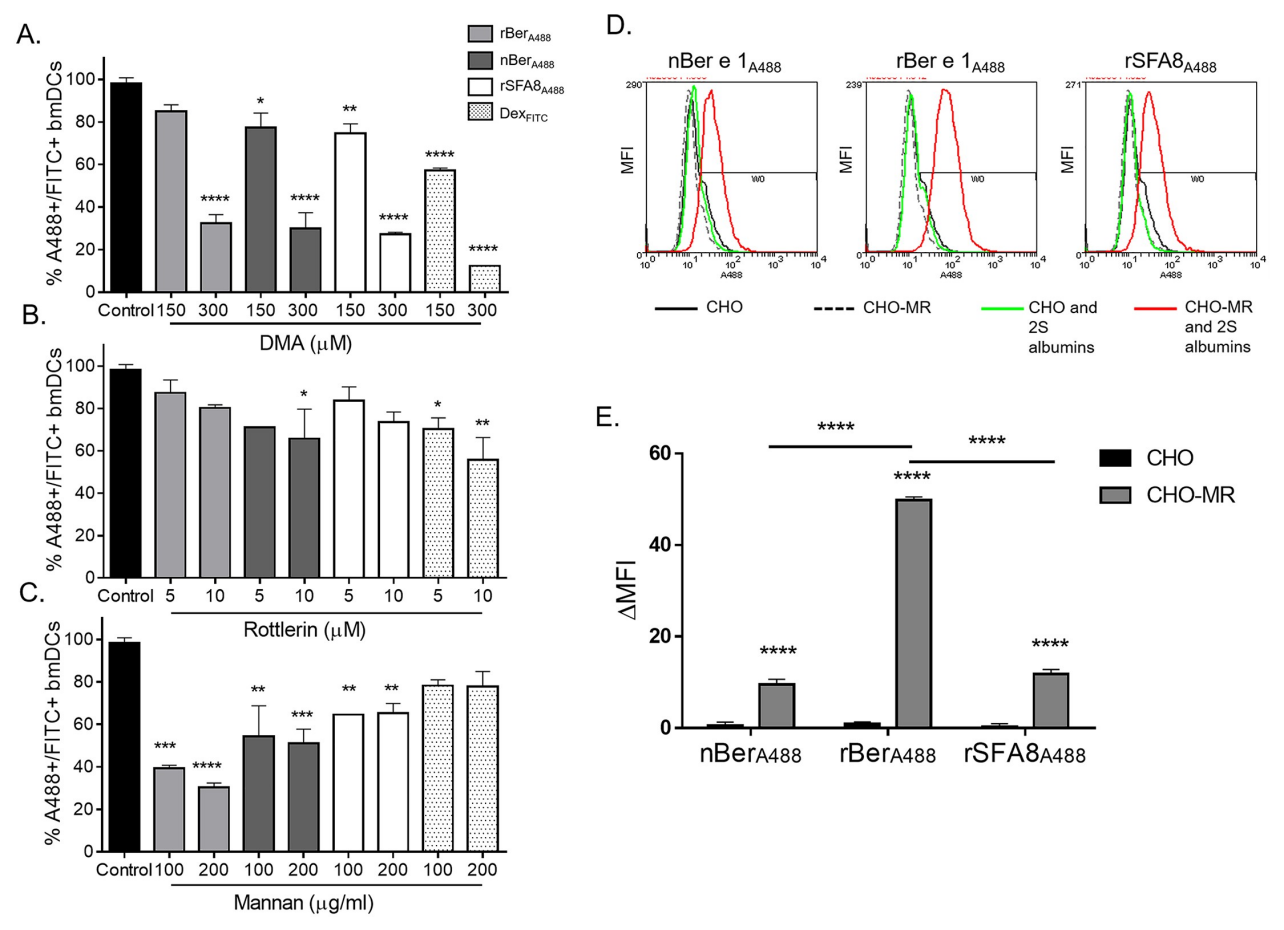
Mechanisms of endocytosis of 2S albumins in bmDC and their uptake in CHO and CHO-MR. (A.) The effect of macropinocytosis inhibitors, DMA and (B.) rottlerin, and (C.) mannose competitive inhibitor, mannan, on the endocytosis of rBer e 1-A488, nBer e 1-A488 and rSFA8-A488 into bmDC was determined by flow cytometry. (D.) The flow cytometry histogram of uptake of A488-labelled proteins by CHO and CHO-MR cell line, as analysed using flow cytometry (E.) The graph showed differences in the uptake of fluorescent-labelled rBer e 1, nBer e 1 and rSFA8 by CHO and CHO-MR where data are presented as normalised median fluorescent intensity (MFI) of cells pulsed with labelled proteins to unpulsed cells and presented as mean ±SD. ** p<0.01, *** p<0.001, ****p<0.0001. Statistical differences are relative to the untreated control.

The endocytosis of water-soluble antigens by immature DC can take place via two main pathways, either via a non-specific fluid-phase endocytosis (macropinocytosis) or clathrin-dependent receptor-mediated endocytosis [37, 38]. Both pathways can be employed interchangeably by immature DC [38]. In order to identify the mechanisms that mediate the endocytosis the binding and uptake of fluorescent-labelled rBer, nBer e 1 and rSFA8 by bmDC were compared by FACs analysis under specific drug inhibitory conditions. Two commonly used inhibitors of macropinocytosis, 5-(N,N-dimethyl)amiloride (DMA) and rottlerin were used. DMA, an analogue of amilorides, inhibits the Na^+^/H^+^ ion exchange pump in the plasma membrane, which affects the intracellular pH. This disruption results in the cessation of the macropinocytosis [39]. The mechanism of rottlerin in inhibiting macropinocytosis is not yet clearly defined although it has been suggested that it inhibits protein kinase C delta (PKCδ) indirectly by uncoupling mitochondria, thus reducing cellular ATP levels (as reviewed by Soltoff, 2007 [40]). Dextran (MW 70,000 Da) was used in this study as a macropinocytic marker, as has been used in other studies [41–43]. Fig 4 showed that the inhibition of protein uptake by DMA and rottlerin was dose dependent using the concentrations previously described in the literature [39, 44, 45]. In the presence of 150 μM DMA, significant reduction was observed in the uptake of rBer e 1, nBer e 1 and rSFA8 (Fig 4A). Internalization of rBer e 1-A488, nBer e 1-A488, rSFA8-A488 and Dextran-FITC was further inhibited at 300 mM DMA by more than 60%.

Whether the uptake of glycosylated rBer e 1 and non-glycosylated nBer e 1 by bmDC is mediated by mannose-specific CLRs was then determined by pre-incubating the cells with different concentrations of mannan from *S*. *cerevisiae*. The process is carried out for 30 min, and further 45 min was spent to pulse with rBer e 1-A488, nBer e 1-A488, or rSFA8-A488 for a further 45 min. The polysaccharide mannan consists of multiple mannose residues that have a higher affinity binding to most mannose-specific CLRs than monomeric glycoproteins and was shown to inhibit CLR-type receptors more efficiently than other sugars [37]. As expected, mannose-specific CLRs were involved in mediating the uptake of rBer e 1 by BmDC; pre-incubation of bmDC with 100 μg/ml and 200μg/ml mannan significantly reduced the amount of glycosylated rBer e 1 taken up by bmDC by 60% and 70% respectively (Fig 4C). Less expected however was the significant drop of approximately 30–40% on the uptake of nBer e 1 and rSFA8. Significant reduction of the uptake of Dextran-FITC was also observed in bmDC in the presence of mannan in other studies [38, 43]. Therefore, these inhibition studies together suggest that both forms of Ber e 1 and rSFA8 entered bmDC via energy-dependent endocytosis. While both forms of Ber e 1 and rSFA8 were taken up by bmDC via a combination of both macropinocytosis and receptor-mediated endocytosis, the bmDC uptake of rBer e 1 was further enhanced via CLR-mediated endocytosis, thus explaining its increased rate of uptake compared to the non-glycosylated nBer e 1 and rSFA8.

### Role of mannose receptor in assisting binding and uptake of 2S albumin proteins

Mannose receptor (MR), a CLR, has been shown to play a role in increasing the immunogenicity of glycoantigens by allowing continuous uptake and accumulation of antigens intracellularly [15, 37, 46]. MR has been found to be highly expressed by immature DC [47]. In order to assess whether glycosylated Ber e 1 is recognised by MR, Chinese hamster ovarian (CHO) cell line constitutively expressing murine MR(CHO-MR) were used. As shown in Fig 4D and 4E, all labelled proteins were internalised by CHO-MR cells, but not by control CHO cells. The uptake of glycosylated rBer e 1 by CHO-MR, which is determined by measuring the change of MFI, was five-fold higher than the uptake of nBer e 1-A488 and rSFA8-A488 (Fig 3E). This suggests that the uptake of A488-rBer e 1 is assisted by MR binding to the mannose residues attached to the recombinant Ber e 1. There also seemed to be some specific uptake of the native proteins nBer e 1 and rSFA8 by CHO-MR cells, which is consistent with the mannan inhibition results (Fig 3C).

### Colocalization of 2S albumin proteins within bmDC

**Fig 5:**
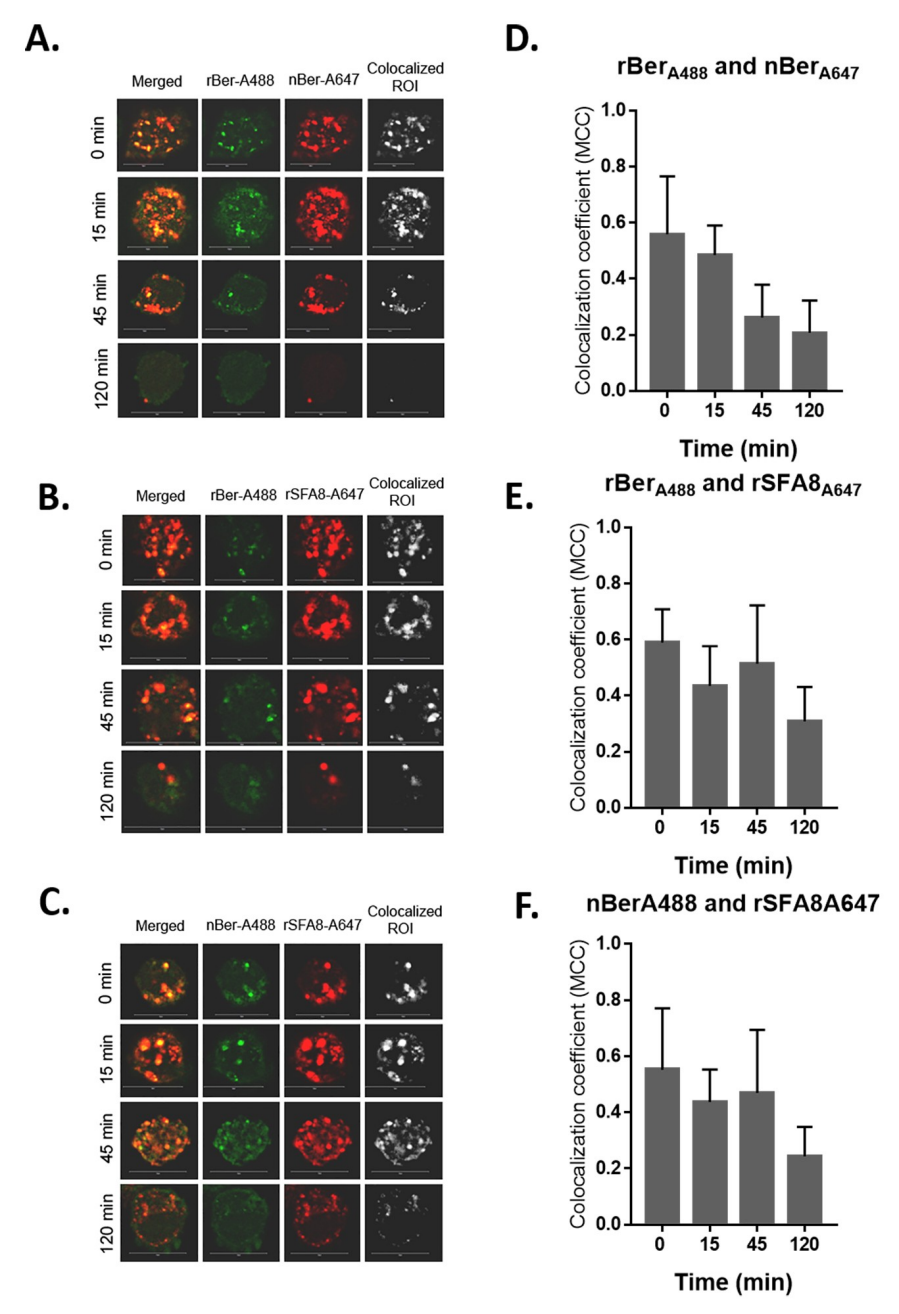
Confocal fluorescence imaging of CD11c^+^ bmDC for colocalization analysis. The colocalization between (A.) rBer e 1-A488 (green) and nBer e 1-A647 (red), (B.) rBer e 1-A488 (green) and rSFA8-A647 (red) and (C.) rBer e 1-A488 (green) and rSFA8-A647 (red), were visualised at different chasing times (0 min, 15 min, 45 min, and 120 min). Gradual reduction of fluorescent-labelled rBer e 1, nBer e 1, and rSFA8 in bmDC were observed over time. Bright spots of colocalized ROI represent areas where both proteins co-occurred. Scale bar for all images = 10 μm. Colocalization after different chasing times were compared for co-occurred regions representing overlapped of the two fluorescent labelled proteins in puncta and measured as MCC. MCC for fractional overlap of A488-labelled proteins and A647-labelled proteins were compared in graphs (D.–F.).

Previous studies suggest that the specific internalisation route of signalling receptor can lead to distinct signalling outcomes [48, 49]. It was hypothesised that the glycosylation of the rBer e 1 will result in distinct routing through the endocytic pathway in bmDC compared to nBer e1 and rSFA8. To test this hypothesis, the confocal analysis of bmDC after internalisation of fluorophore-labelled proteins was carried out (Fig 5A–5C), and the degree of colocalization was determined by calculating the Mander’s colocalization coefficient (MCC). Upon initial co-introduction of both rBer e 1-A488 and nBer e 1-A647 to the bmDC (t = 0 min), the MCC value of 0.557 ±0.208 indicates that about 56% rBer e 1 colocalized with nBer e 1 at distinct puncta. After 15 minutes, the MCC values dropped slightly to 0.48 ±0.11 (Fig 5D). Post 45 to 120 minutes of chasing time, the MCC values of colocalization between rBer e 1 and nBer e 1 were reduced to 0.260 ±0.119 and 0.206±0.116, respectively (Fig 5D). Similarly, for rBer e1 and rSFA8 (Fig 5E), the MCC values dropped to 0.470 ±0.224 and 0.244 ±0.104, respectively. Based from this result, we suggest that the majority of the proteins were either degraded or released unprocessed back to the extracellular environment after 120 minutes, as previously reported for proteins internalised via macropinocytosis and proteins colocalized in macropinosomes [43, 50]. However, we could not confirm this suggestion as we did not measure the protein content level in the medium. Interestingly, for non-glycosylated nBer e 1 and rSFA8, both proteins remained colocalised at consistent MCC values from 0 to 45 minutes, (0.590 ±0.208 and 0.513 ±0.122 respectively) but dropped to 0.308 ±0.122 after 120 minutes.

### Glycosylation of recombinant Ber e 1 did not stimulate the maturation of bmDC and induced lower Th1 and Th2 polarising cytokines

**Fig 6:**
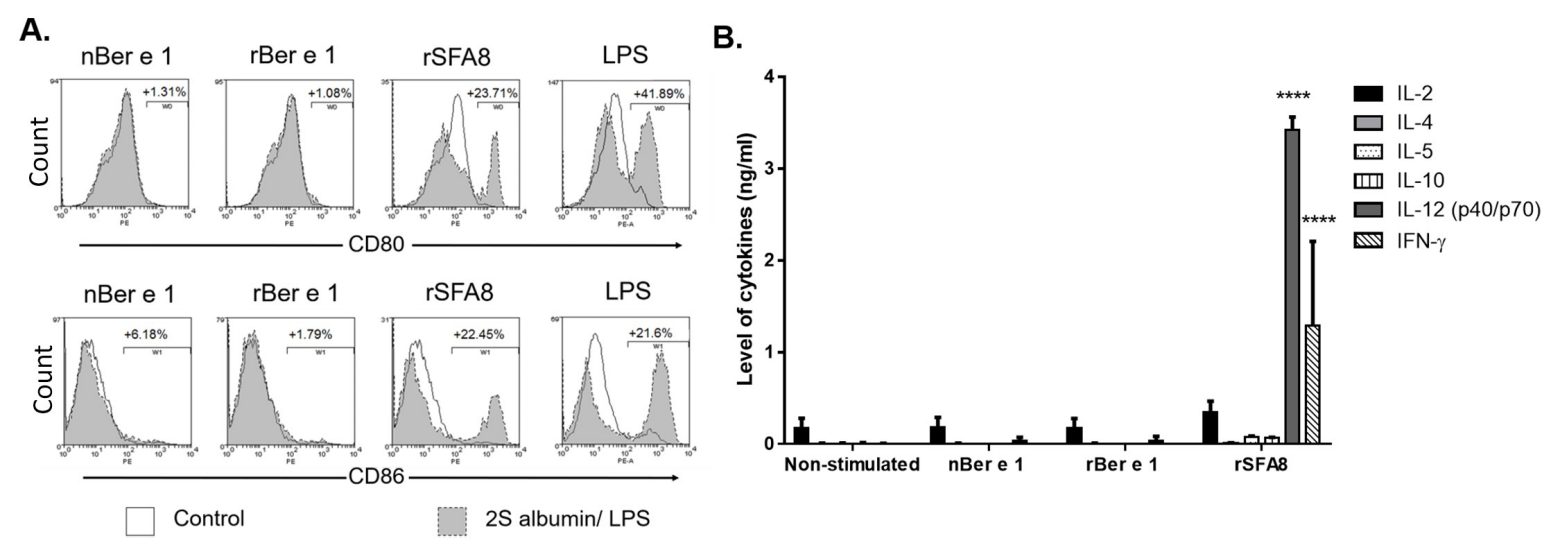
Co-stimulatory molecular markers and Th1/Th2 cytokine production upon CD11c^+^ bmDC stimulation with 2S albumins. (A.) Percentage population of CD80^+^ or CD86^+^ bmDC post stimulation with 50 μg/ml rBer e 1, nBer e 1, or rSFA8, or 10 ng/mL LPS (positive control) were normalised to non-stimulated bmDC. (B.) Level of Th1 (IL-12, and IFN-γ)—and Th2 (IL-2, IL-4, IL-5)-specific cytokines detected in bmDC stimulated with 50 μg/ml of nBer e 1, rBer e 1 rSFA8 or 10 ng/ml LPS were measured after 24 hours by Luminex assay. Data are from three biological replicates and presented as mean ± SD (****, p < 0.0001) relative to non-stimulated bmDC.

Upon bmDC internalisation of proteins, different signals were internally activated that would alter the cell maturation state [51]. Thus, in order to assess the phenotypically maturation state of bmDC upon pulsing with rBer e 1, nBer e 1 and rSFA8 after 24 hours, two classical markers of maturation (CD80 and 86) were monitored (Fig 6A). CD80 and CD86 are co-stimulatory molecules that are highly expressed in mature DC [51, 52]. A clear shift in the MFI of LPS-induced bmDC when compared to non-stimulated bmDC revealed that the CD80 and CD86 were highly presented on the cell surface. The low levels of CD86 and CD80 on bmDC stimulated with rBer e 1 and nBer e 1 were equivalent to the non-stimulated BmDC, revealing that the cells remained in their immature state. Stimulation with rSFA8 resulted in marginal increases in CD80 and CD86, indicating some DC maturation but at a lower degree than when stimulated with LPS.

In order to assess the internal signals produced upon internalization, six common cytokines involved in classical Th1/Th2 responses were measured (Fig 6B). Cytokine levels were consistent with previous findings [53]. Stimulation of bmDC with rBer e 1 and nBer e 1 did not elicit production of either Th1-specific cytokines or Th2-specific cytokines. However, unexpectedly, the rSFA8-induced bmDC produced significant amounts of Th1-specific cytokines, IL-12, Interferon gamma (IFN-γ) and TNFα [53]. We have shown that the endotoxin levels detected in our samples, rBer e 1, nBer e 1, and rSFA8, were very low at less than 0.006 EU/μg (S1 Table). IL-2, IL-4 and IL-5 were not detected in all samples. Interferon gamma (IFN-γ) is generally produced by natural killer (NK) and T cells and less commonly by CD11c^+^ DC. However, DC have also been shown to be able to produce IFN-γ upon stimulation and IL-12 administration via autocrine positive feedback pathways [54, 55]. Anti-inflammatory cytokine, IL-10, was not detected in bmDC stimulated with any of the proteins tested. Overall, these data are in agreement with previous 2S albumin-DC-T cell polarisation findings [53].

## Discussion

2S albumins are normally expressed in multigene families and are found *in planta* as a mixture of isoforms, some proteolytically processed into small and large subunits, that retain similar protein folding and three-dimensional structures. In order to produce a consistent homogeneous population of proteins, we have expressed rBer e 1 and SFA8 as secreted proteins in the *Pichia pastoris* system. These proteins were highly purified, correctly folded and have been shown to share similar physicochemical properties and identical immunoreactivity to their native counterparts [16, 26]. By exchanging protein domains between SFA8 and Ber e 1, chimeric proteins had been produced that helped to map the hypervariable loop regions involved in IgE recognition [16]. The proteolytic processing of SFA8 and the putative presence of short *O*-linked glycans on the rBer e 1 as opposed to its native counterpart had also been reported [16, 26, 53]. In this study, we addressed the differences between *P*. *pastoris*-derived recombinant Ber e 1 and its native counterparts in terms of glycosylation, and determined whether the fungal glycosylation of the recombinant proteins could affect the immunogenicity of the recombinant proteins, which may in turn affect the signal transduction process involved in the modulation of immunity. It has been suggested that the presence of glycans on allergens is important during sensitisation as they participate in their recognition, uptake, and presentation by antigen presenting cells [19]. In this context glycosylation has been shown to play important role as allergenic determinants in well characterised allergens such as Ara h 1 from peanut (*Arachis hypogaea*) and Der p 1 from house dust mites (*Dermatophagoides pteronyssinus*) amongst others [22].

Like all members of the prolamin group (Pfam, Pfam.sanger.ac.uk), Ber e 1 is known to have four anti-parallel bundles of α-helices, bridged by four disulphide bonds between cysteine residues on large and small chains (two interlinked and two intralinked disulphide bonds), and has been shown to possess a very rigid and compact structure which is resistant to proteolytic digestion [16, 23]. The rBer e 1 protein is not easily linearised by the reducing agent such as DTT due to its highly compact structure, high methionine contents, and the presence of *O-*linked glycosylation which increase the stability of the protein structure [37, 56]. These findings explained why we detected peptide sequences from small and large subunits of Ber e 1 from both 15kDa gel bands of rBer e 1 and nBer e 1 samples. The compact 3D structure with its high content of methionine interlinked by the disulphide bridges bestow an almost impenetrable barrier for treatments, such as enzymatic/chemical deglycosylation, pepsin digestion and treatment with chaotropic reagents, which can be detected in circular dichroism analysis even at high temperatures [23].

Detection of glycan residues on protein by SDS-PAGE—PAS staining revealed and confirmed our previous findings that only *P*. *pastoris*-derived rBer e 1 is glycosylated but not rSFA8 or their wild type counterparts nBer e 1 and nSFA [16]. Detection of trypsin-digested glycopeptides by ESI-q-ToF II MS further support previous NMR and mass spectrometric data [16, 26]. Specifically, it showed that the *O*-linked glycosylation on rBer e 1 is heterogeneous relative to the number of glycans attached and that the attachment sites predominantly occur on the large polypeptide chain. Here it is shown that the glycosylation occurs at Ser96, Ser101 and Ser110 on the large chain and Ser19 on the small polypeptide chain. The heterogeneity of *O*-linked glycosylation on rBer e 1 did not seem to affect the protein folding processes, as rBer e 1 was correctly folded as shown in previous structure studies [16, 23, 26]. This contrasts with other findings which showed that the fungal glycosylation by *P*. *pastoris* resulted in the improper protein folding of recombinant Der p 1 which required further de-glycosylation for protein maturation [14].

The effect of fungal *O*-linked glycosylation on the *in vitro* antigenicity of the rBer e 1 was further investigated, using a DC-T cell assay and PBMCs from healthy human donors with diverse HLA backgrounds using a commercial ProImmune REVEAL™ Immunogenicity System. Genetic background has been shown to modulate immune responses, for example, specific HLA haplotypes resulted in predisposed individuals to develop specific antibody response against particular products [57]. In ProImmune REVEAL healthy donors were HLA haplotype mapped and selected to represent global HLA allele frequencies. An increase in T cell proliferation from donors with different HLA alleles was observed between glycosylated rBer e 1 and non-glycosylated nBer e 1. Although both forms were poor immunogens with very low RIs compared to positive controls, the RI for rBer e 1 was two-fold higher than for nBer e 1. These results suggest that the glycosylation on rBer e 1 has a small but measurable effect on the *in vitro* antigenicity of the protein in comparison to its non-glycosylated counterpart nBer e 1. Similarly, it has been also shown by others that the short *O*-linked glycans present in *P*. *pastoris*-derived recombinant OVA facilitated protein uptake by murine bmDC and increased the proliferation of CD4^+^ T cells than its non-glycosylated counterparts [15, 58].

The commercial HLA human platform is based on healthy donors from diverse genetic backgrounds. Thus, in order to reduce the impact of heterogeneous genetic background, a murine *in vitro* DC model system was employed to analyse the interaction of the different allergen preparations with DC. In this study we used female BALB/c mice as they have been shown to be more susceptible to elicit allergic reaction than male mice [59–61]. The immunogenicity of an antigen is generally regarded as a result of a sequence of events such as antigen uptake, DC activation, processing and stability of MHC class II-peptide complex presentation [62, 63]. In this context, the recognition and engagement of glycans to specific CLR-lectins may elicit different intracellular and extracellular responses that lead to different signalling pathways. Engagement of glycoproteins to MR for instance has been shown to activate expression of an anti-inflammatory cytokine, IL-10. An inhibitory effect of mannan-rich lipoarabinomannans engaging to MR has been observed on the production of IL-12 by DC during the Th2 polarisation [64]. In the system described here it has been demonstrated that CLR-mediated endocytosis was involved in the uptake of glycosylated rBer e 1, based on the inhibitory effect of mannan on the cellular uptake. The binding of rBer e 1 to murine MR expressed on CHO cell line was five-fold higher than that of the non-glycosylated nBer e 1 and rSFA8. Yet here we also observed low levels of uptake of nBer e 1 and rSFA8 by CHO-MR cells in spite of non-existent glycosylation in both proteins, as determined by the PAS-stained gel (Fig 1), MS analysis (Table 1), and our previous NMR findings [26]. MR, as a C-type lectin carbohydrate-binding receptor, is commonly expressed on most macrophages, DC, and epithelial cells [65]. It is known be involved in the uptake of glycoconjugates by binding the glycan- (mannose-, fucose-, or N-Acetylglucosamine) terminal of the glycoconjugates to its C-type lectin-like domains via a calcium dependent process [65, 66]. The macrophage MR has been shown previously to be able to recognise collagens independent of carbohydrates by binding the collagens to the MR fibronectin-type II domain [67]. Based from our preliminary finding here, we therefore speculate that the MR may potentially recognise the non-glycosylated 2S albumin proteins independent of the sugar residues.

The results presented here also demonstrated that the glycosylation of rBer e 1 increases the rate of antigen uptake by DC compared to non-glycosylated Ber e 1 and rSFA8. However, the uptake by bmDC of fluorescent-labelled rBer e 1, nBer e 1 and rSFA8 were shown as a combination of both non-specific macropinocytosis and receptor-mediated endocytosis. Following previous studies that showed a Th1/Th2 polarisation of Ber and SFA8 proteins in the murine system [43], the sharing of the same uptake system as determined by confocal analysis was unexpected. These proteins showed a moderately high degree of colocalization between the labelled proteins at the early time points (0 to 45 min) in undetermined cellular compartments. These findings were in contrast to those observed in microbial and helminth products with polarising Th1/Th2 properties, where each was taken up by DC via distinct intracellular compartments [68].

DC are key players in the induction of immune responses, and their function is closely influenced by their level of maturation. Maturation of DC is known to be mediated by different danger signals such as microbial products, and results in upregulated expression of co-stimulatory molecules. To assess the effects of rBer e 1, nBer e 1 and rSFA8 on bmDC, molecular maturation markers were measured after 24 hours of post stimulation. The results showed that neither glycosylated rBer e 1 nor non-glycosylated nBer e 1 induced or altered the maturation of bmDC, as frequencies of cells expressing CD80 and CD86 remained unaffected, in stark contrast to rSFA8. The non-conventional effect of rSFA8 was previously described on bmDC [43], and it was not due to the endotoxin contamination as all protein preparations were shown to have negligible traces of endotoxins (S1 Table), or driven by TLR4 as MyD88 knock out were equally responsive [53].

The observed absence of Th1-polarising IL-12 and the immature state of murine bmDC when stimulated with either rBer e 1 or nBer e 1, are in agreement with our previous findings [53]. This observation also agrees with the “default hypothesis for Th2 immunity” described by several authors [69, 70]. There are other endocytic pathways that were not explored in this study but might also be involved in the uptake of the 2S albumin model allergens. For instance, recent studies by Smole *et al*. demonstrated that Bet v 1 uptake by monocyte-derived DC was impeded by inhibiting caveolae-dependent mechanisms with filipin [71]. Bet v 1, similarly to Ber e 1, possesses a hydrophobic cavity that assists its DC uptake via caveolae-mediated endocytosis by binding to the amphiphilic and lipid ligands, which are the components of lipid raft-dependent caveolae [70]. Caution should be exercised as some of the currently available commercial pharmacological drugs or biological inhibitors have been shown to cause pleiotropic effects that may affect different endocytic mechanisms. This effects may also be cytotoxic to cells, as observed when inhibiting potential clathrin-mediated endocytosis by hypertonic sucrose inhibition and chlorpromazine [72]. While we managed to demonstrate here that the fungal glycosylation of the Ber e 1 increased its *in vitro* antigenicity, the results presented here, however, further support previous data where Ber e 1 alone, regardless of its glycosylation status, is a poor immunogen and incapable of stimulating allergenic reaction during sensitisation, and may require other natural compounds present in the nut matrix to stimulate sensitisation [73].

## Conclusions

Taken together, the results suggest that the glycans attached on rBer e 1 have a small but measurable effect on the *in vitro* antigenicity of the protein in comparison to its non-glycosylated counterpart, nBer e 1. The glycosylation of rBer e 1 also introduced possible new epitopes for HLA class II binding compared to nBer e 1 as determined by the variable CD4^+^ T cell proliferation responses of different HLA-specific individuals. Glycans present on *P*. *pastoris*-produced rBer e 1 increased the efficiency of the protein recognition and internalization by bmDC via the MR compared to its non-glycosylated counterpart native Ber e 1 and the weak allergenic rSFA8, similar to other findings [15]. Engagement of glycosylated rBer e 1 to MR alone did not induce the production of IL-10, which modulates the DCs to polarise the Th2 cell response by suppressing the IL-12 production and DC maturation. While this study indicated possible similar routes of internalization of rBer e 1, nBer e 1, and rSFA8 in bmDC at specific times, further studies on the differential modulation of murine bmDC upon stimulation with either Ber e 1 and SFA8, and comparison of their molecular mechanisms are warranted.

## Supporting information

S1 Fig Graphs show the DRB1 (A), DQB1 (B) and DPB1 (C) allele frequency distribution of the donor panel selected for commercial human DC-T cell assay compared to the distribution in the global population (ProImmune, Oxford, UK).
(DOCX)

S2 Fig The histograms represent the percentage population of GM-CSF-dependent CD11b^+^ and CD11c^+^ bmDCs, as determined by the flow cytometry. (DOCX)

S3 Fig Raw images of (A) PAS-stained (used in Fig 1A) and (B) Commassie-stained (used in Fig 1B and 1C) 12% BisTris NuPAGE gel under denaturing conditions (300 mM DTT), where (1) positive glycoprotein control, CPDY; (2) rSFA8; (3) rBer e 1; and (4) nBer e 1. Images of both PAS- and Coomassie-stained gels were captured on Gel Doc XR+ System (BioRad, USA). ‘X’ lanes represent empty lanes and lanes that were loaded with rSFA8 samples before undergoing RP-HPLC purification, flow through of RP-HPLC, and nSFA that were not included in Fig 1A, 1B and 1C.
(PDF)

S1 Table Level of endotoxin detected in 2S albumin proteins used in this study. (DOCX)

S2 Table Panels of healthy human donors selected predominantly to represent the HLA class II alleles that are known to be highly expressed in the global population (ProImmune, Oxford, UK). (DOCX)

S1 File (XLSX)
