# Supplementary material for: Effect of O-linked glycosylation on the antigenicity, cellular uptake and trafficking in dendritic cells of recombinant Ber e 1
Source: PLoS One. 2021 Apr 29;16(4):e0249876. doi: 10.1371/journal.pone.0249876 (PMC8084162; doi:10.1371/journal.pone.0249876)
Supplement: S1 Fig — Graphs show the DRB1 (A), DQB1 (B) and DPB1 (C) allele frequency distribution of the donor panel selected for commercial human DC-T cell assay compared to the distribution in the global population (ProImmune, Oxford, UK). (DOCX) [file pone.0249876.s001.docx]

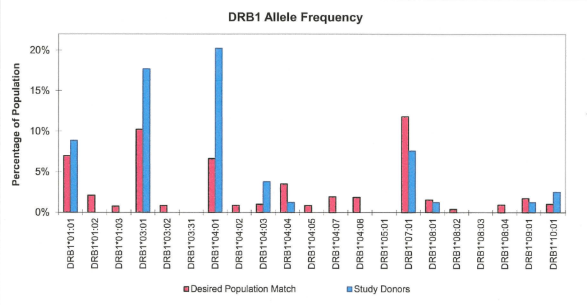


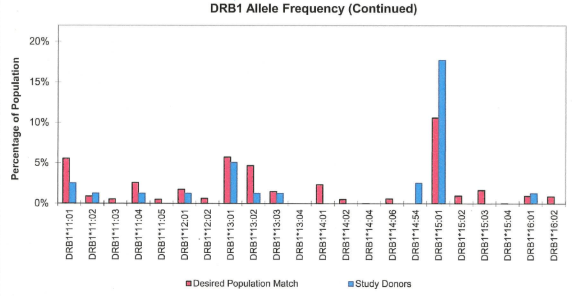


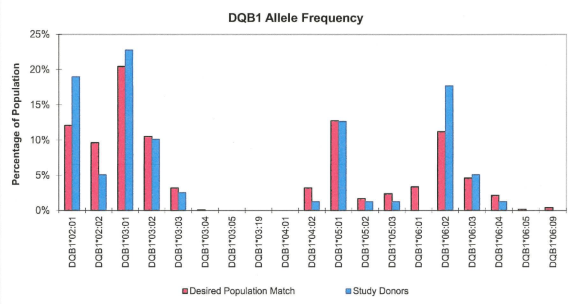


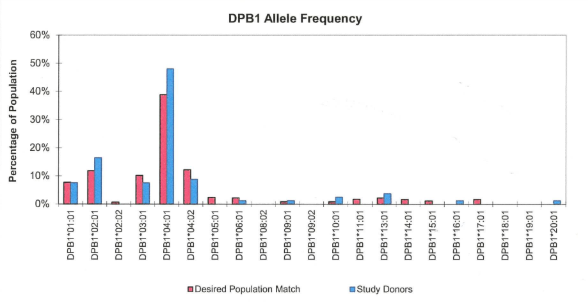


**S1** **Fig .** Graphs show the DRB1 (A), DQB1 (B) and DPB1 (C) allele frequency distribution of the donor panel selected for commercial human DC-T cell assay compared to the distribution in the global population (ProImmune, Oxford, UK).
