# Supplementary material for: Effect of O-linked glycosylation on the antigenicity, cellular uptake and trafficking in dendritic cells of recombinant Ber e 1
Source: PLoS One. 2021 Apr 29;16(4):e0249876. doi: 10.1371/journal.pone.0249876 (PMC8084162; doi:10.1371/journal.pone.0249876)
Supplement: S2 Fig — (DOCX) [file pone.0249876.s002.docx]

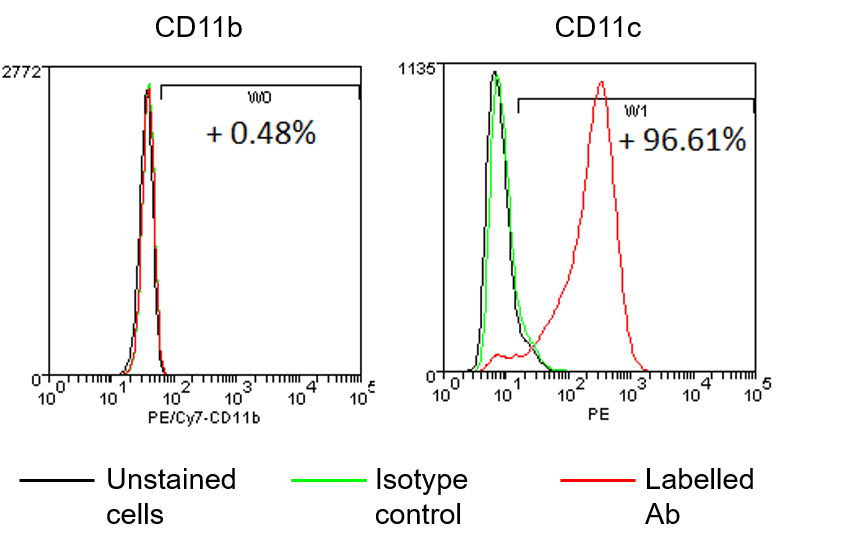


**S2 Fig. The histograms represent the percentage population of GM-CSF-dependent CD11b^+^ and CD11c^+^ bmDCs, as determined by the flow cytometry.**
