# Supplementary material for: Effect of O-linked glycosylation on the antigenicity, cellular uptake and trafficking in dendritic cells of recombinant Ber e 1
Source: PLoS One. 2021 Apr 29;16(4):e0249876. doi: 10.1371/journal.pone.0249876 (PMC8084162; doi:10.1371/journal.pone.0249876)
Supplement: S3 Fig — Raw images of (A) PAS-stained (used in Fig 1A) and (B) Commassie-stained (used in Fig 1B and 1C) 12% BisTris NuPAGE gel under denaturing conditions (300 mM DTT), where (1) positive glycoprotein control, CPDY; (2) rSFA8; (3) rBer e 1; and (4) nBer e 1. Images of both PAS- and Coomassie-stained gels were captured on Gel Doc XR+ System (BioRad, USA). ‘X’ lanes represent empty lanes and lanes that were loaded with rSFA8 samples before undergoing RP-HPLC purification, flow through of RP-HPLC, and nSFA that were not included in Fig 1A, 1B and 1C. (PDF) [file pone.0249876.s003.pdf]

A.

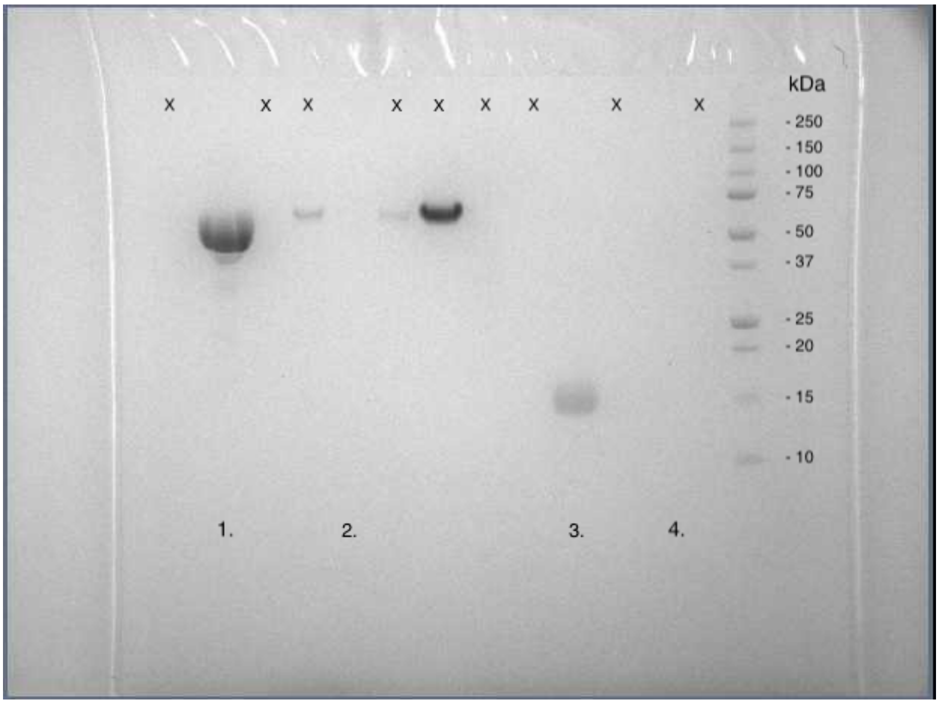

B.

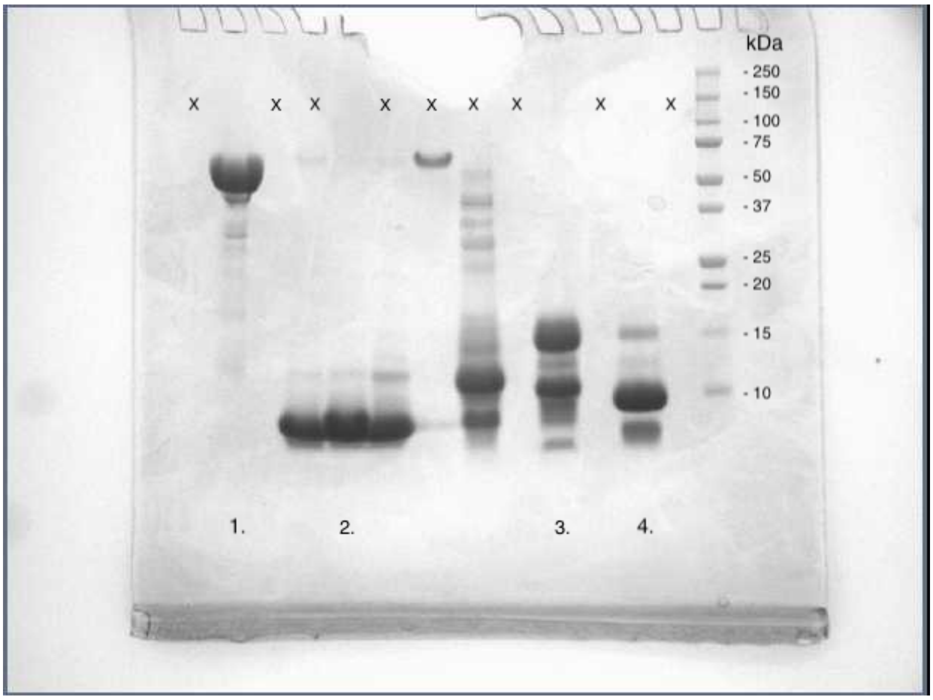

S3 Fig. Raw images of (A.) PAS-stained (used in fig. 1A.) and (B.) Commassie-stained (used in Fig. 1B. and 1C.) 12% BisTris NuPAGE gel under denaturing conditions (300 mM DTT), where (1.) positive glycoprotein control, CPDY; (2.) rSFA8; (3.) rBer e 1; and (4.) nBer e 1. Images of both PAS- and Coomassie-stained gels were captured on Gel Doc XR+ System (Bio-Rad, USA). 'X' lanes represent empty lanes and lanes that were loaded with rSFA8 samples before undergoing RP-HPLC purification, flow through of RP-HPLC, and nSFA that were not included in fig. 1A., 1B. and 1C.
