# Supplementary material for: Effect of O-linked glycosylation on the antigenicity, cellular uptake and trafficking in dendritic cells of recombinant Ber e 1
Source: PLoS One. 2021 Apr 29;16(4):e0249876. doi: 10.1371/journal.pone.0249876 (PMC8084162; doi:10.1371/journal.pone.0249876)
Supplement: S1 Table — (DOCX) [file pone.0249876.s004.docx]

**S1 Table.** Level of endotoxin detected in 2S albumin proteins used in this study

| 2S albumin protein | Endotoxin level (EU/µg) | S.D. |
| --- | --- | --- |
| nBer e 1 | 0.006168 | ±0.000516 |
| rBer e 1 | 0.006018 | ±0.002816 |
| rSFA8 | 0.004170 | ±0.000768 |
