# Supplementary material for: Effect of O-linked glycosylation on the antigenicity, cellular uptake and trafficking in dendritic cells of recombinant Ber e 1
Source: PLoS One. 2021 Apr 29;16(4):e0249876. doi: 10.1371/journal.pone.0249876 (PMC8084162; doi:10.1371/journal.pone.0249876)
Supplement: S2 Table — (DOCX) [file pone.0249876.s005.docx]

**S2** **Table.** Panels of healthy human donors selected predominantly to represent the HLA class II alleles that are known to be highly expressed in the global population (ProImmune, Oxford, UK)

| **Donor ID** | **DRB1_1** | **DRB1_2** | **DQB1_1** | **DQB1_2** | **DPB1_1** | **DPB1_2** |
| --- | --- | --- | --- | --- | --- | --- |
| D318 | *04:01 | *07:01 | *02:02 | *03:01 | *04:01 | *02:01 |
| D510 | *01:01 | *04:01 | *03:01 | *05:01 | *04:01 | *04:01 |
| D532 | *04:01 | *15:01 | *03:01 | *06:02 | *04:01 | *04:01 |
| D544 | *04:01 | *04:01 | *03:01 | *03:01 | *04:01 | *04:02 |
| D547 | *03:01 | *03:01 | *02:01 | *02:01 | *04:01 | *04:01 |
| D574^ǂ^ | *01:01 | *03:01 | *02:01 | *05:01 | *04:01 | *04:02 |
| D583 | *04:01 | *04:01 | *03:02 | *03:01 | *04:01 | *04:01 |
| D597 | *01:01 | *15:01 | *05:01 | *06:02 | *04:01 | *04:02 |
| D598 | *03:01 | *04:01 | *02:01 | *03:02 | *02:01 | *02:01 |
| D610 | *01:01 | *04:01 | *03:01 | *05:01 | *04:01 | *04:01 |
| D619 | *13:02 | *15:01 | *06:04 | *06:02 | *02:01 | *01:01 |
| D620 | *03:01 | *12:01 | *02:01 | *03:01 | *02:01 | *01:01 |
| D621 | *03:01 | *04:01 | *02:01 | *03:02 | *01:01 | *04:02 |
| D631 | *03:01 | *04:04 | *02:01 | *03:02 | *06:01 | *04:01 |
| D651 | *03:01 | *04:01 | *03:01 | *02:01 | *02:01 | *03:01 |
| D655 | *01:01 | *13:01 | *05:01 | *06:03 | *04:01 | *10:01 |
| D783 | *03:01 | *07:01 | *02:02 | *02:01 | *02:01 | *09:01 |
| D784 | *04:03 | *13:01 | *06:03 | *03:02 | *04:01 | *03:01 |
| D788 | *04:01 | *15:01 | *03:01 | *06:02 | *04:01 | *04:01 |
| D790 | *04:03 | *13:01 | *06:03 | *03:02 | *16:01 | *03:01 |
| D792 | *07:01 | *07:01 | *02:02 | *02:01 | *02:01 | *13:01 |
| D793 | *07:01 | *13:03 | *02:02 | *03:01 | *04:01 | *03:01 |
| D797 | *10:01 | *16:01 | *05:01 | *05:01 | *10:01 | *02:01 |
| D798 | *10:01 | *11:01 | *03:01 | *05:01 | *13:01 | *04:01 |
| D803 | *03:01 | *15:01 | *02:01 | *06:02 | *04:01 | *02:01 |
| D806 | *04:01 | *14:54 | *05:02 | *03:01 | *04:01 | *02:01 |
| D816 | *03:01 | *11:04 | *02:01 | *03:01 | *04:01 | *04:02 |
| D818 | *07:01 | *15:01 | *06:02 | *03:03 | *04:01 | *04:01 |
| D819 | *03:01 | *15:01 | *02:01 | *06:02 | *13:01 | *01:01 |
| D820 | *03:01 | *15:01 | *02:01 | *06:02 | *20:01 | *01:01 |
| D822 | *15:01 | *15:01 | *06:02 | *06:02 | *04:01 | *04:01 |
| D823 | *04:01 | *08:01 | *04:02 | *03:01 | *04:01 | *02:01 |
| D829 | *04:01 | *09:01 | *03:03 | *03:01 | *04:01 | *03:01 |
| D831 | *11:02 | *15:01 | *03:01 | *06:02 | *04:01 | *04:02 |
| D835 | *15:01 | *11:01 | *03:01 | *06:02 | *04:01 | *01:01 |
| D840 | *03:01 | *15:01 | *02:01 | *06:02 | *04:01 | *04:01 |
| D841 | *04:03 | *15:01 | *06:02 | *03:02 | *02:01 | *02:01 |
| D843 | *01:01 | *13:01 | *05:01 | *06:03 | *04:01 | *04:01 |
| D846 | *01:01 | *14:54 | *05:01 | *05:03 | *04:01 | *03:01 |
| D848 | *04:01 | *04:01 | *03:02 | *03:02 | *04:01 | *04:02 |
